# Potentially toxic elements in wild Agassiz’s desert tortoises: tissue concentrations and association with disease

**DOI:** 10.3389/fvets.2024.1481367

**Published:** 2024-11-21

**Authors:** Kristin H. Berry, Mary M. Christopher, Elliott R. Jacobson

**Affiliations:** ^1^U.S. Geological Survey, Western Ecological Research Center, Reno, NV, United States; ^2^Department of Pathology, Microbiology and Immunology, School of Veterinary Medicine, University of California, Davis, Davis, CA, United States; ^3^Department of Small Animal Clinical Sciences, College of Veterinary Medicine, University of Florida, Gainesville, FL, United States

**Keywords:** disease, environmental contamination, *Gopherus agassizii*, heavy metals, macrominerals, trace minerals, Mojave Desert, Colorado Desert

## Abstract

**Background:**

Desert tortoise (*Gopherus agassizii*) populations have continued to decline due to infectious and other diseases, predation, and habitat alteration. The potential contribution of minerals and heavy metals to tortoise health and susceptibility to disease remains uncertain.

**Objective:**

The objective of this study was to evaluate the results of elemental analysis on trace minerals, macrominerals, and heavy metals in scute keratin, kidney, and liver from ill and dying desert tortoises salvaged for necropsy between 1993 and 2000.

**Methods:**

Salvaged tortoises were categorized by size (adult, juvenile), geographic location, and primary disease based on necropsy findings. A subset of tortoises that were injured or killed by vehicular trauma or predation but with no notable pathologic abnormalities was used for comparison with diseased tortoises. The panel of elements was analyzed in scute keratin, kidney, and liver samples by inductively-coupled plasma spectrometry and atomic absorption spectrophotometry.

**Results:**

Necropsies were done on 46 tortoises, including 9 juveniles, salvaged from 5 regions in the Colorado and Mojave Deserts of California. Primary diseases were cutaneous dyskeratosis (*n* = 9), infection/inflammation (*n* = 8), malnutrition (*n* = 7), mycoplasmosis (*n* = 5), and urolithiasis (*n* = 3); 14 tortoises died of trauma. Concentrations of elements differed by tissue, size, desert region, and disease status (*p* < 0.05). Tortoises with cutaneous dyskeratosis had higher Se concentrations, primarily in keratin and liver, than tortoises with other diseases (*p* < 0.001). Juveniles were more likely than adults to have high Pb, Sn, and Zn levels (*p* < 0.05). All tortoises had detectable levels of more than one potentially toxic heavy metal, including As, Cd, Cr, Hg, Ni, Pb, Sn, and V.

**Conclusion:**

Potentially toxic elements are frequently found in tissues from tortoises in desert regions of California, with higher concentrations in diseased tortoises. Metal exposure from soils, mining, historic and ongoing military activities, and other human activities could increase susceptibility to disease in desert tortoises.

## 1 Introduction

Agassiz’s desert tortoise (*Gopherus agassizii*, herein desert tortoise or tortoise) is a federally threatened species throughout the geographic range, which is north and west of the Grand Canyon-Colorado River complex (1). The geographic range includes the deserts of southeastern California, southern Nevada, northwestern Arizona, and the extreme southwestern corner of Utah in the United States. Since the federal listings in 1990, populations have continued to decline due to infectious and other diseases, hyper-predation by avian and canid predators, and multiple anthropogenic alterations to their habitats, causing loss, degradation, and fragmentation (2–4). Allison and McLuckie (2) reported that the “species is on the path to extinction under current conditions.” The tortoise was listed as critically endangered in 2021 on the International Union of Conservation of Nature’s Red List (5), and uplisted from threatened to endangered status in California in 2024 (6).^1^

The human footprint looms large for the desert tortoise in the American Southwest (7). Tortoise habitats, including designated critical habitat units, are degraded and fragmented by agricultural and urban development; grazing of livestock and feral burros; mining and industrial developments (e.g., cement plants, incinerators); roads, railroads, petroleum and gas pipelines and utility corridors; military activities; vehicle-oriented recreation; invasion of non-native annual grasses and forbs; and more recently by energy development (1, 3, 8–13). Uptake of toxic elements by tortoises may occur from contact with soil, breathing dust, eating soil and rocks at water catchments and mineral licks, and eating plants with contaminated dust caught in the pubescence and glands of plant parts (14–17). Tortoises have limited sizes of home ranges (e.g., 17–47 ha) with the ranges of adult males typically larger than those of females or juveniles [e.g., (18, 19)]. Tortoises exhibit fidelity to burrows and home sites [e.g. (20, 21)]. Therefore, uptake of heavy metals or other elements is likely limited to the home range or slightly beyond. Wind may carry contaminants in disturbed soils from considerable distances, however. Uptake of elemental toxicants will depend on location, diet, and size of the tortoise, and historical and ongoing anthropogenic changes to habitat.

The tortoise is particularly vulnerable to human activities and disturbances to habitats because of life history characteristics: high mortality in early life stages, 17 to 20 or more years to attain sexual maturity, and even then, females may lay few eggs until reaching 30 or more years of age (21–24). Life spans are estimated at >80 years when tortoises live in relatively undisturbed habitats (21, 25).

The species lives primarily in the Mojave and Colorado (the western region of the Sonoran Desert) deserts, where precipitation is low and unpredictable with frequent droughts (26). The tortoise has several adaptations to cope with the harsh desert climate, but these adaptations add to its vulnerability. Tortoises spend much of life underground (>95%), avoiding temperature extremes of winter and summer and lack of food and water, thereby lowering metabolic rates during winter brumation and summer estivation (27, 28). Importantly, drought affects reproductive viability. Females may resorb yolks, lay no eggs, or produce smaller clutches (24, 29–31). All tortoises, but especially smaller individuals, may succumb to malnutrition, dehydration, and starvation (32–34).

The periodic droughts followed by precipitation and food availability are potential pathways for uptake and retention of toxic elements in tissues.When rain comes or follows a period of drought, tortoises respond rapidly to drinking water at self-dug catchments or where water collects (17, 28). Regardless of body size, when rain breaks a drought, tortoises can drink copious amounts of water, replacing up to 40% of body weight lost during drought (22, 35). Winter rains stimulate germination and growth of annual forbs and herbaceous perennial species favored by the herbivorous tortoises [e.g., (36, 37)]. During rain events, tortoises void wastes stored in the bladder, and refill the bladder with dilute fluid that can be resorbed (28, 35). During droughts, tortoises tolerate high concentrations of sodium, chloride, potassium, and urea in plasma and the same electrolytes can become isosmotic with bladder fluid (35). Potassium, which is potentially toxic in plasma, is excreted as fluid potassium and potassium urate to the bladder (35). During and after a rain, drinking, and voiding the bladder, potassium is excreted, avoiding the toxic effects of hyperkalemia (28, 35). Tortoises have adapted to the threat of hyperkalemia through selection of plants low in potassium as forage (37–39). During periods of drought, elemental toxicants may become concentrated in internal organs.

New and emerging diseases also present important challenges to the viability of desert tortoise populations and were one of several factors contributing to the federal listing in 1990 (1). An upper respiratory tract disease caused by *Mycoplasma agassizii* was first described from the field in 1989 (40) and later named in 2001 (41). *M. agassizii* caused clinical signs and contributed to chronic illness and widespread death in some areas (42–44). *Mycoplasma testudineum,* another upper respiratory tract infection described in 2004, caused similar clinical signs but appeared to be less pathogenic (45, 46). Herpesvirus is a third pathogen that can cause upper respiratory signs and lesions in the oral cavities of desert tortoises (47). Upper respiratory tract diseases in Testudinids were summarized by Jacobson et al. (44).

Cutaneous dyskeratosis, an apparent non-infectious disease, also contributed to the federal listing in 1990 (1). This disease, prevalent since 1988 in tortoises on the Chuckwalla Bench in the Colorado Desert and since diagnosed widely, especially in the Fenner Valley in the eastern Mojave Desert, causes characteristic lesions and flaking of scutes, and, in severe cases, loss of scutes, necrosis of bones, and effects in other organs (48, 49). While its etiology is unknown, potential causes are nutritional deficiencies (e.g., vitamin A) and exposure to elemental toxicants, including Se and heavy metals. Bacteria and fungi may be present but appear to be secondary invaders (48). Cutaneous dyskeratosis has some similarities with necrotizing scute disease associated with fungi in the Texas tortoise, *G. berlandieri* (50) and with shell degradation in gopher tortoises, *G. polyphemus* (51), but the lesions are for the most part distinct in desert tortoises.

Chelonians have essential requirements for some elements and not for others [e.g., (52)]. Minerals can be essential nutrients required for biological processes. Trace minerals are those required in only small amounts, e.g., Cr, Co, Cu, Fe, Mn, Mo, Se, and Zn; whereas macrominerals, e.g., Ca, Mg, P, K, and Na, are required in relatively high amounts. Trace heavy metals, e.g., Al, Cd, Hg, Ni, Pb, Sn, and V are potentially toxic elements that may or may not be required. Trace elements can become harmful to health in elevated concentrations and can serve as biomarkers of environmental contamination. To our knowledge, it is not known which heavy metals may be required in trace amounts and which are potentially toxic in desert tortoises.

Studies of potentially toxic elements in desert tortoises are limited, despite increasing research on the ecotoxicology of reptiles over the decades [e.g., (52–54)]. Most research has occurred on sea turtles and aquatic turtles. A search of the literature using the genera of tortoises revealed a few studies on tortoises, including one on Pb toxicity in gopher tortoises (*Gopherus polyphemus*) on a military reservation (55). In Agassiz’s desert tortoises in the Mojave desert of California, higher levels of Fe, Hg, and Pb were reported in livers of ill tortoises with mycoplasmosis compared with healthy tortoises (40). Arsenic was linked to shell and respiratory disease in tortoises (48, 49). More recently, laser ablation with inductively coupled plasma mass spectrometry (ICP-MS) techniques demonstrated localized high concentrations of As in scutes of 4 tortoises and uptake of As in specific growth rings or laminae of ill tortoises compared with those without underlying disease (56). Valence and distribution of As in scute and lung tissue of healthy and ill necropsied tortoises were determined using X-ray absorption fine structure spectroscopy (15). Finally, Cohn et al. (57) evaluated 5 heavy metals in 191 tortoises using dried blood spots from populations in the northeast region of the Mojave Desert and reported that metal concentrations rarely exceeded minimum detection levels.

The objective of this observational, largely descriptive study was to evaluate the results of selected trace elements, macrominerals, and heavy metals in scute, kidney, and liver tissues from ill and dead desert tortoises salvaged for necropsy examination. Gross and histopathologic findings, together in some cases with clinical and laboratory findings, were used to assess health status and establish the primary disease. We hypothesized that concentrations of elements would differ among types of tissues, size groups, desert regions, and diseases. We also identified tortoises with one or more outlying values and with detectable values of potentially toxic elements of high concern. We discuss the potential sources of trace elements in the environment. These results will likely enhance our understanding of the prevalence of trace minerals in desert tortoise tissues in different regional habitats and their association with size and disease. These findings will improve our ability to identify potentially toxic elements that warrant further investigation regarding tortoise morbidity and mortality.

## 2 Materials and methods

### 2.1 Data collection on tortoises

**Figure 1:**
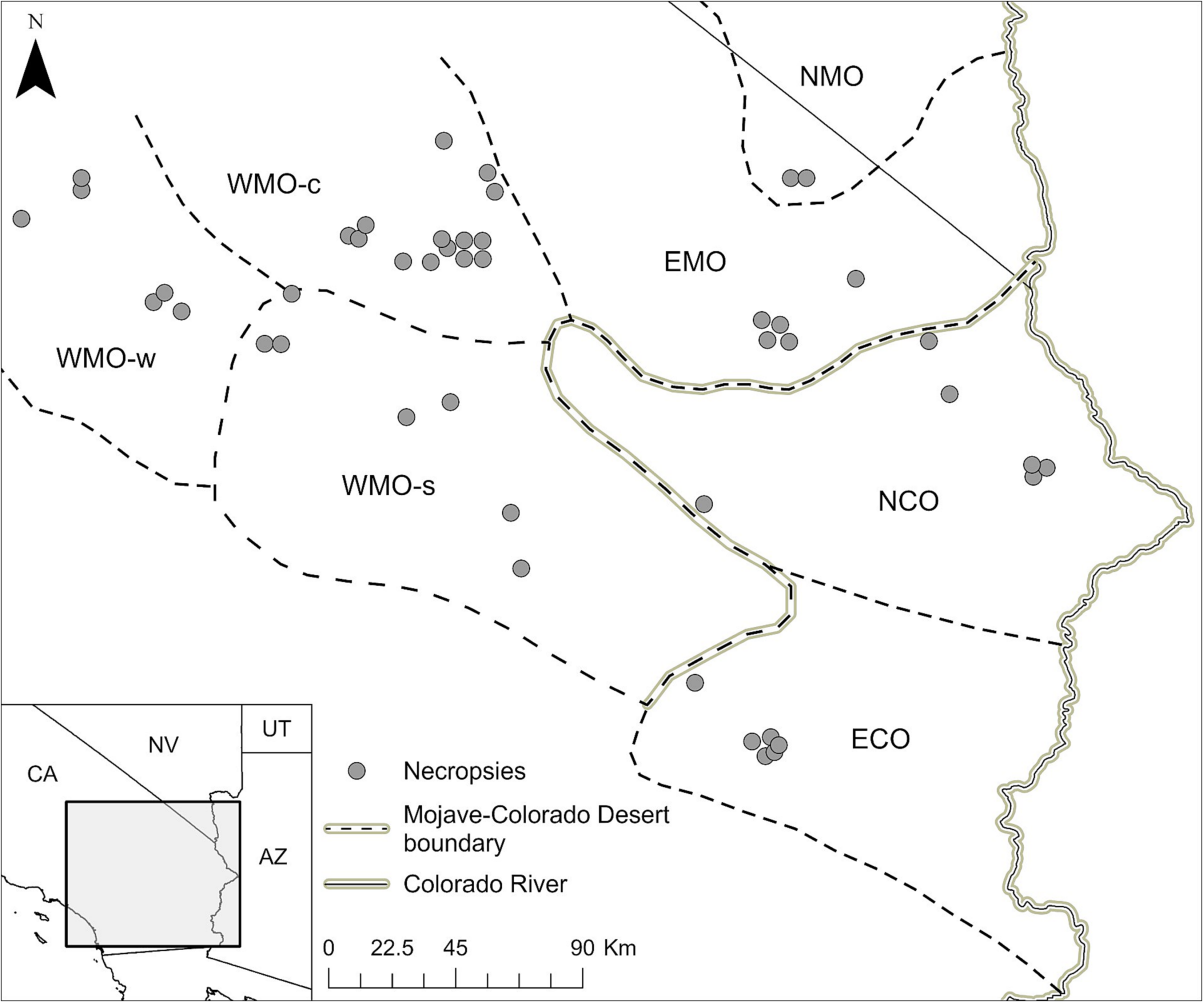
Location and distribution of salvaged Agassiz’s desert tortoises (*Gopherus agassizii*) in regions of the Mojave and Colorado (western Sonoran) deserts of California, United States. The regions are WMO-w, Western Mojave-western section; WMO-c, Western Mojave-central section; WMO-s, Western Mojave-southern section; EMO, East Mojave section; NMO, northeastern Mojave section; NCO, Northern Colorado section; ECO, Eastern Colorado section. NCO and ECO were combined in the analyses as COLO. EMO and NMO were combined in the analyses as EMO.

Clinically ill, moribund, and freshly dead (no obvious degradation or putrefaction) desert tortoises were salvaged for necropsy under state and federal permits from the California Department of Fish and Game [Permit No. SC-003623] and U.S. Fish and Wildlife Service [Permit No. TE-06556 and amendments], respectively, to KHB. All animal procedures were approved by the Western Ecological Research Center, U.S. Geological Survey’s Animal Care and Use Committee. Necropsies of tortoises were approved by the University of Florida’s Institutional Animal Care and Use Committee (Protocol A769). All tortoises were found in the Mojave and Colorado deserts of California (Figure 1).

Tortoises were shipped alive in specially constructed wooden boxes via air freight, or freshly dead on ice, or frozen to the University of Florida at Gainesville, United States, for necropsy examinations by a board-certified veterinary pathologist (48). Live tortoises were humanely euthanized by barbiturate overdose prior to necropsy. The pathologic findings, disease processes (including results of bacterial and fungal culture), and presumptive cause of death for 24 of the tortoises (48) and another 11 tortoises (32) were previously reported. Additional tortoises were salvaged through 2000, bringing the total reported here to 46 (Supplementary Table S1).

Data obtained during tortoise collection and necropsy were analyzed, including: date of collection, site of collection, sex, size as determined by metric measurements of body length (straight-line carapace length at the midline, MCL, mm; adults, ≥180 mm; juveniles, <180 mm), body weight (BW, kg), primary disease, and other disease processes. The pathologist’s interpretation of results for laboratory tests for *Mycoplasma* sp., complete blood counts, and biochemical profiles were available for most tortoises salvaged when alive. Not all tests were done in all tortoises, and histologic examination of tissues in tortoises found dead was sometimes limited because of autolysis or freezing prior to transfer to the necropsy facility. Based on our review of the available clinical, necropsy and laboratory data for each tortoise, the primary cause of death was categorized as mycoplasmosis (positive serology and/or culture for *Mycoplasma* sp.; clinical and/or laboratory evidence for rhinitis or pneumonia); cutaneous dyskeratosis (shell or cutaneous lesions of flaking, discoloration, peeling lamina, scute defects, superficial or minor fungal or bacterial colonization without tissue inflammation); infection/inflammation (inflammatory cell infiltrates of shell, epithelium or internal organs; systemic leukocytoses, with or without intralesional bacteria or fungi); malnutrition (severe emaciation; atrophy of fat and internal organs, osteopenia, hypoalbuminemia, hypovitaminoses); and urolithiasis (obstructive urolith in bladder; clinical and laboratory abnormalities associated with severe dehydration). Several tortoises had multiple disease conditions but were categorized based on the primary disease at the time of salvage. Tortoises that were recently killed by vehicles or predators and that had no significant pathologic abnormalities at necropsy were categorized as” trauma,” for the purpose of comparison with diseased tortoises.

### 2.2 Elemental analysis

During necropsy, samples of scute keratin, liver, and kidney were collected for analysis using metal-free tools. Scutes were cleaned prior to sampling. Samples were sent to the United States Department of Agriculture’s National Veterinary Services Laboratories in Ames, Iowa, United States, where they were analyzed for 27 elements. Five elements were macrominerals (Ca, K, Mg, Na, and P), 9 were trace minerals (Cr, Co, Cu, Fe, Mn, Mo, S, Se, and Zn), and the remaining 13 were trace metals (Ag, Al, As, Au, Ba, B, Cd, Hg, Ni, Pb, Sn, Tl, V). Of those tested, Ag, Al, Au, Ba, Cd, Hg, Ni, Pb, Sn, Tl, and V were considered nonessential for reptiles but have not been defined for desert tortoises [e.g., (52)]. The Laboratory followed standard operating and quality control procedures (protocol No. TCPR00103). All elements except Se, Hg, Cu, and Zn, were analyzed by ICP-MS [PerkinElmer 6,500 spectrophotometer (Waltham, MA, United States) with a Teledyne CETAC™ Technologies U-5000 ultrasonic nebulizer (Omaha, NE, United States)]. Se was determined using a gas liquid chromatograph equipped with electron capture detector, auto sampler and suitable column, and a PerkinElmer Integrator Model 1,020 Personal Integrator. Hg, Cu, and Zn were analyzed using an atomic absorption spectrophotometer (PerkinElmer 5,000) equipped with a nebulizer assembly and 10 cm air/acetylene burner head [Technicon AutoAnalyzer II and Integrator (SEAL Analytical, Mequon, WI, United States)]; PerkinElmer Nelson Model 1,022 LC Plus and Terminal Computer set in terminal mode and connected to a data handler/controller. Standard reference materials (elements) from the National Institute of Standards and Technology^2^ were analyzed concurrently for calibration and to establish reporting limits. The reporting limit, as defined by the American Industrial Hygiene Association Laboratory Accreditation Program^3^ is “the lowest concentration of analyte in a sample that can be reported with a defined, reproducible level of certainty” and is based on the concentration of the lowest standard reference material.

Not all analyses were done on all tissues, depending on tissue availability and volume. Results of most analytes were expressed as parts per million (ppm) on a wet weight basis. S concentration in keratin samples was expressed as a percentage. When analyte concentration was nondetectable (below the lowest standard reference material), results were reported as less than the reporting limit (e.g., <0.10 ppm).

### 2.3 Statistical analysis

Carapace length and BW were reported as mean ± standard deviation (SD). The 7 desert regions where tortoises were salvaged were grouped into 4 regions based on geographic proximity and tortoise genetic similarity (58) as follows: West Mojave-western and central sections (WMO-WC), West Mojave-southern section (WMO-S); East and Northeast Mojave (EMO), and Northern and Eastern Colorado (COLO) (Figure 1).

All statistical analyses were done using JMP®Pro 17.0.0 (JMP Statistical Discovery LLC, Cary, NC, United States). For the purpose of analysis, nondetectable results were assigned a value of one-half the reporting limit. The results of elemental analysis were highly skewed, both with and without log-transformation, based on visual observation and Shapiro–Wilk tests. Therefore, results were reported as the median (minimum-maximum) and nonparametric statistical tests were used to compare groups. Wilcoxon rank sum (2-sample) or Kruskal-Wallis tests with post-hoc Wilcoxon tests were used to compare analytical results based on tissue, size group, sex, region, and disease group. Statistical significance was defined as *p* < 0.05.

The proportion of nondetectable results for all elements by tissue was determined. Outliers were identified (but not removed from the dataset) by examination of box and whisker plots for each element and tissue in which values above or below the end of the whiskers were outliers >1.5X the interquartile range (1.5 X IQR rule). Detectable concentrations of elements for which ≥90% of total results were nondetectable were also considered to be outliers. These rare detectable outliers together with outliers found above the upper end of the whiskers for other elements were considered to be “high” values for a given element.

## 3 Results

### 3.1 Metrics of tortoises, regions, and diseases

**Figure 2:**
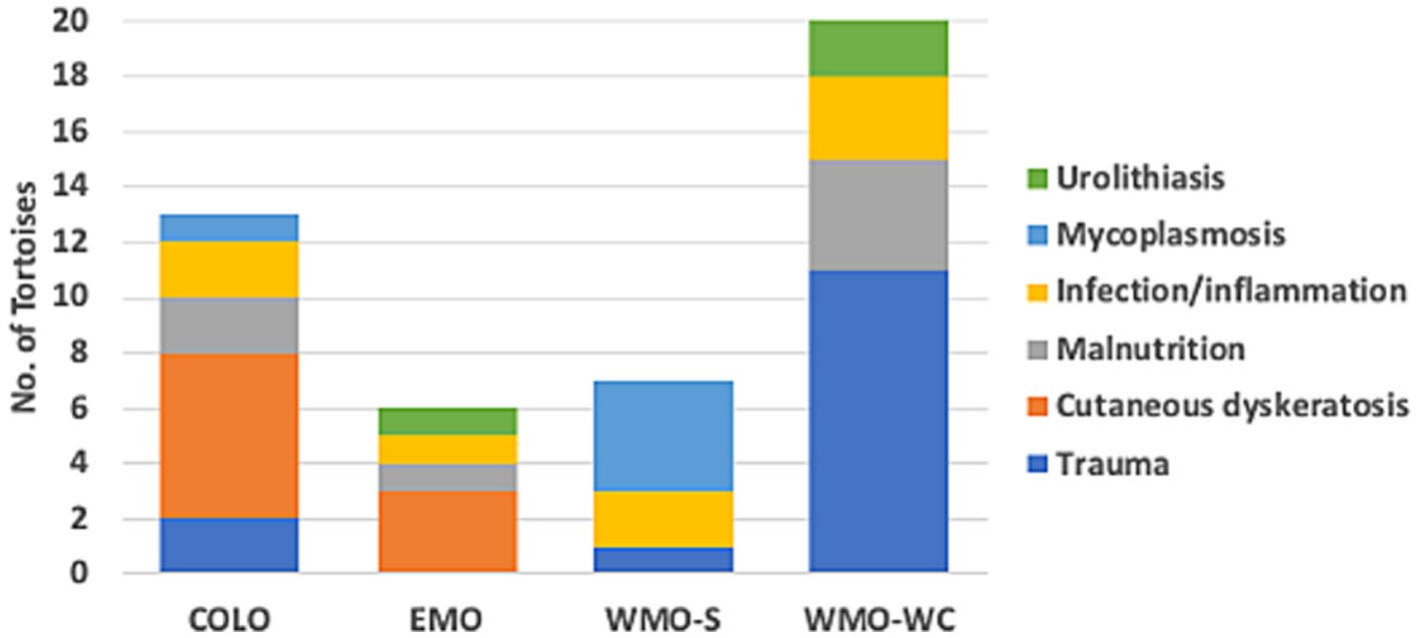
Diseases identified in necropsies of 46 Agassiz’s desert tortoises (*Gopherus agassizii*) in regions of the Mojave and Colorado (western Sonoran) deserts of California, United States. Tortoises with trauma died primarily from collision with vehicles and, in one case, predation.

Forty-six tortoises were necropsied between 1993 and 2000 (Supplementary Table S1). The tortoises were primarily adults (*n* = 37, 80.4%) with fewer juveniles (*n* = 9, 19.6%) and included both males (*n* = 27, 58.7%) and females (*n* = 17, 40.0%). The sex of two juvenile tortoises was not reported. Mean (± SD) BW was 1.9 ± 1.3 kg (adults, 2.3 ± 1.1 kg; juveniles, 0.5 ± 0.3 kg); BW was not reported for one adult male tortoise. Mean (± SD) MCL was 206.5 ± 66.6 mm (adults, 232.7 ± 36.7 mm; juveniles, 98.4 ± 50.3 mm). The tortoises were salvaged from the COLO (10 adults, 3 juveniles), EMO (5 adults, 1 juvenile), WMO-S (7 adults), and WMO-WC (15 adults, 5 juveniles) desert regions (Figures 1, 2).

Fourteen tortoises found dead had injuries consistent with acute trauma —killed by vehicular trauma (9 hit by military vehicle, 4 hit by car) or acute predation (1)—and otherwise had no pathologic abnormalities at necropsy. Three of these tortoises had evidence of autolysis at necropsy, which limited histologic examination, but the evidence of trauma (e.g., overturned, fractures and dislocation, protruding viscera, ruptured spleen) confirmed the cause of death. The remaining tortoises had necropsy and histopathologic evidence of disease, which was categorized as follows: cutaneous dyskeratosis, infection/inflammation, malnutrition, mycoplasmosis, and urolithiasis (Table 1). Mycoplasmosis is also an infectious disease with inflammation but was considered separately because of its importance in desert tortoise populations (42, 44). Of the 9 tortoises with cutaneous dyskeratosis, 3 also had infection/inflammation, 1 had malnutrition, and 5 had loss of condition or suspected nutritional deficiencies (Supplementary Table S1). Of the 7 tortoises with malnutrition, 6 (85.7%) were juveniles. Of 8 tortoises with infection/inflammation, 4 also had cutaneous dyskeratosis and 1 had malnutrition. One tortoise with urolithiasis also had evidence of renal disease. A higher number of tortoises in the COLO and EMO regions had cutaneous dyskeratosis, in the WMO-S region had mycoplasmosis, and in the WMO-WC died of trauma (Figure 2).

**Table 1:** Primary disease process in 46 Agassiz’s desert tortoises (*Gopherus agassizii*) salvaged from the Mojave and Colorado deserts of California, United States, between 1997 and 2000 and based on primary disease categories.

Primary disease	Primary necropsy findings	No. (%) of tortoises
Cutaneous dyskeratosis	White, flaky, discolored shell lesions, cracks and fissures, with altered stratum corneum, epithelial atrophy, dermal irregularity, osteoclastic resorption ± necrosis or organisms	9 (19.5)
Infection/inflammation	Heterophilic (± lymphocytic) inflammation in tissue(s) with bacteria and/or fungi ± necrosis and inflammatory leukogram; negative for *Mycoplasma* sp.	8 (17.4)
Malnutrition	Lack of coelomic fat, decreased muscle mass, osteopenia of shell and long bones, testicular, liver, and skeletal muscle degeneration	7 (15.2)
Mycoplasmosis	Culture and/or serology positive for *Mycoplasma* sp.; clinical and histologic signs of upper respiratory tract disease ± inflammatory leukogram	5 (10.9)
Trauma	Fractures and traumatic injuries consistent with being struck by vehicle or predator attack	14 (30.4)
Urolithiasis	Large urolith in bladder ± renal tubular necrosis with intralesional urate crystals ± cystitis	3 (6.5)

### 3.2 Elemental analyses

A total of 2,848 analyses were completed, of which 1866 had measurable concentrations and 976 (34.3%) were nondetectable. Seventeen analyses had missing results due to insufficient sample mass. More than 50% of all results for Ag, As, Au, B, Co, Ni, Pb, Sn, and Tl were nondetectable and > 50% of keratin results were nondetectable for Cd, Cr, Cu, Mo, and V (Table 2). Ninety percent or more of As, Au, Ni, Pb, and Sn results and 100% of Ag and Tl results were nondetectable.

**Table 2:** Concentrations of elements in keratin, kidney and liver samples from 46 Agassiz’s desert tortoises (*Gopherus agassizii*) salvaged from the Mojave and Colorado (western Sonoran) deserts of California, United States, between 1993 and 2000*.

Element	No. Samples	% Detectable	Keratin	No. Samples	% Detectable	Kidney	No. Samples	% Detectable	Liver	*p* value†	Test statistic *(H)*†
Ag	37	0	<2.5	27	0	<0.1	31	0	<0.1	—	—
Al	35	100	93 (18–272) ↑	25	96.0	3 (1–204) ↓	23	100	11 (1–370)	<0.001	51.5
As	33	6.1	1.0 (0.1–15.0)	40	2.5	0.5 (0.1–6.0)	40	2.2	0.5 (0.1–3.0)	(<0.001)	(21.0)
Au	38	8.1	0.8 (0.1–5.1)	27	7.4	0.5 (0.1–2.5)	29	10.7	0.5 (0.1–1.8)	(0.234)	(2.9)
Ba	37	89.2	1.9 (0.1–7.9) ↑	37	97.3	0.3 (0.1–3.7)	44	90.7	0.3 (0.1–7.8)	<0.001	32.9
B	19	31.6	2.5 (0.5–9.6)	18	63.2	0.5 (0.5–4.9) ↓	19	63.2	2.5 (0.5–15.4)	0.039	6.5
Ca	37	100	820 (225–5,900) ↑	27	100	135 (46–1,650)	43	100	84 (21–1,600) ↓	<0.001	57.1
Cd	37	29.7	0.1 (0.1–3.6)	40	100	1.3 (0.2–5.3) ↑	46	100	0.3 (0.1–2.3)	<0.001	51.3
Co	37	16.2	0.1 (0.1–2.5)	36	58.3	0.2 (0.1–1.2)	44	33.6	0.1 (0.1–0.3)	(<0.001)	(26.4)
Cr	37	29.7	0.24 (0.03–2.5)	36	58.3	0.15 (0.05–0.66)	44	65.1	0.20 (0.05–7.10)	0.404	1.8
Cu	37	45.9	0.1 (0.1–2.1)	37	94.6	2.0 (0.1–13.0)	46	100	7.0 (0.9–75.0) ↑	<0.001	82.2
Fe	37	100	97 (32–350)	37	100	37 (14–220) ↓	46	100	535 (74–3,300) ↑	<0.001	78.7
Hg	35	82.9	0.03 (0.01–0.50) ↓	31	93.5	0.15 (0.01–1.15)	40	100	0.19 (0.04–0.82) ↑	<0.001	33.6
K	35	100	104 (45–3,803) ↓	26	100	2005 (995–3,000)	21	100	2,100 (1170–2,680)	<0.001	52.4
Mg	37	100	93 (45–250) ↓	37	100	150 (70–470)	43	100	145 (76–380)	<0.001	27.8
Mn	37	100	2.1 (0.6–7.8) ↑	39	100	1.1 (0.4–8.3)	45	100	1.0 (0.2–15.0)	<0.001	41.1
Mo	37	10.8	0.1 (0.1–2.5) ↓	36	91.7	1.1 (0.1–3.6)	44	97.7	1.1 (0.4–5.1)	(<0.001)	(34.5)
Na	37	100	560 (80–1700) ↓	37	100	1,600 (870–4,700)	44	100	1,200 (390–3,700)	<0.001	63.9
Ni	37	10.8	0.5 (0.5–200) ↑	37	5.4	0.25 (0.15–7.5)	44	13.9	0.25 (0.15–7.5)	(<0.001)	(53.9)
P	37	100	335 (42–2000) ↓	37	100	2,300 (1300–3,800)	44	100	2,100 (890–4,000)	<0.001	71.8
Pb	37	8.1	0.50 (0.05–7.5) ↑	40	5.0	0.25 (0.15–3.8)	46	6.7	0.25 (0.15–2.1)	(<0.001)	(37.8)
S	33	100	—	23	100	1750 (1170–2,810)	19	100	1,660 (1090–2,790)	0.206	1.6
Se	37	100	0.2 (0.1–2.4) ↓	14	100	0.7 (0.1–2.0)	39	100	0.4 (0.1–3.5)	0.002	12.8
Sn	19	15.8	0.1 (0.1–9.0) ↑	20	0	0.1 (0.1–0.1)	26	4.0	0.1 (0.1–0.3)	(<0.001)	(50.0)
Tl	19	0	< 15.0	18	0	< 5.0	19	0	< 5.0	—	—
V	35	37.1	0.3 (0.1–2.5)	36	52.8	0.2 (0.1–2.4)	43	71.4	0.3 (0.1–5.1)	0.099	4.6
Zn	37	100	40 (16–84) ↑	40	100	36 (13–230)	45	100	27 (7–140)	0.001	14.2

*Data are median (minimum–maximum) in ppm on a wet weight basis. Results for nondetectable results were calculated as the reporting limit ÷ 2; Ag and Tl had no detectable values so the reporting limit is indicated. Results in shaded cells are significantly higher (yellow) or lower (blue) than those in other tissues for that analyte (Kruskal-Wallis with post-hoc Wilcoxon tests). †Values in parentheses may indicate trends, but otherwise should be interpreted cautiously as sample groups for all tissues (As, Au, Ni, Pb, Sn) or for keratin (Co, Mo) have >80% nondetectable values. — indicates statistical testing was not done (no detectable values).

Significant differences in elemental concentrations were observed among tissues (Table 2), with concentrations of Al, Ba, Ca, Mn, and Zn higher in keratin than in kidney and liver. Cd was higher in kidney, and Cu, Fe and Hg concentrations were higher in liver. Adult tortoises had higher concentrations of Cd in kidney and Al, Mo, and V in liver compared with juvenile tortoises (Table 3). Element concentrations did not differ based on sex. Tortoises in the EMO region had significantly higher Se and Cu concentrations than in other regions (Table 4; Supplementary Table S2). Tortoises with trauma were more prevalent in the WMO-WC region (Figure 2) and had lower concentrations of several elements in liver (Tables 4, 5). Tortoises with cutaneous dyskeratosis had significantly higher Se concentrations in keratin and liver; those with malnutrition (primarily juveniles) had higher Zn concentrations in kidney; and tortoises with urolithiasis had higher Cd concentrations in liver (Table 5; Supplementary Table S3). Diseased tortoises as a group had significantly higher concentrations of several elements compared with tortoises that died of trauma (Table 6). Only Ca and Fe in kidney were significantly lower in diseased tortoises than those with trauma.

**Table 3:** Significant differences in concentrations of elements based on size in Agassiz’s desert tortoises (*Gopherus agassizii*) salvaged from the Mojave and Colorado (western Sonoran) deserts of California, United States.*

Tissue	Element	N	Adults	N	Juveniles	*P* value†	Test statistic (*S*)
Kidney	Cd	34	1.3 (0.2–5.3)	6	0.5 (0.3–1.0)	0.043	69
Liver	Al	20	11 (1–370)	3	4 (1–6)	0.040	13
	Mo	35	1.3 (0.4–5.1)	7	0.7 (0.5–1.7)	0.041	92
	V	35	0.5 (0.1–5.1)	7	0.1 (0.1–0.3)	0.002	61

*Data are median (minimum-maximum) in ppm on a wet weight basis. †Wilcoxon 2-sample test. Significant age differences were not observed in keratin samples. Elements with >50% nondetectable results were excluded from analysis.

**Table 4:** Significant differences in concentrations of elements in Agassiz’s desert tortoises (*Gopherus agassizii*) based on region in the Mojave and Colorado (western Sonoran) deserts, United States.*

Tissue	Element	*N*	Colorado Desert	*N*	East Mojave Desert	*N*	West Mojave-S	*N*	West Mojave-WC	*P* value	Test statistic *(H)*
Keratin	Hg	10	0.05 (0.02–0.50)	4	0.02 (0.02–0.02) ↓	7	0.03 (0.03–0.04)	14	0.03 (0.01–0.26)	0.036	8.6
	Na	10	428 (220–750)	4	132 (117–805)	7	465 (133–870)	16	678 (80–1700) ↑	0.023	9.6
	Se	10	0.3 (0.1–1.8)	4	1.7 (0.3–2.4) ↑	7	0.2 (0.1–1.1)	16	0.2 (0.1–0.3)	0.004	13.1
Kidney	Cd	12	1.2 (0.5–3.9)	5	3.8 (0.8–5.3)	7	1.8 (0.5–5.2)	16	0.4 (0.2–3.0) ↓	0.002	15.3
	Cu	12	2.2 (0.1–13.0)	4	4.0 (2.9–11.0) ↑	7	2.3 (1.6–4.4)	14	1.6 (0.1–6.6)	0.012	10.9
Liver	Cd	13	0.4 (0.1–1.2)	6	0.8 (0.2–0.9)	7	0.9 (0.2–1.8)	19	0.3 (0.1–2.3) ↓	0.014	10.7
	Fe	13	535 (190–1,450)	6	1,150 (650–1,500)	7	1,300 (250–3,300)	19	330 (74–1800) ↓	0.004	13.3
	K	7	2,350 (1370–2,680)	3	2,180 (2010–2,250)	3	2,100 (1390–2,140)	8	1,495 (1170–2,250) ↓	0.019	10.0
	Mo	13	1.1 (0.5–2.4)	5	2.1 (0.7–5.1)	7	2.3 (0.9–5.0)	18	1.1 (0.4–2.8) ↓	0.021	9.7
	Se	12	0.4 (0.2–3.5)	5	0.7 (0.4–2.0) ↑	7	0.6 (0.2–1.5)	14	0.3 (0.1–0.6) ↓	0.016	10.3
	V	13	0.2 (0.1–2.0)	5	0.6 (0.2–0.9)	6	1.3 (0.2–5.1)	18	0.2 (0.1–3.7) ↓	0.033	8.7

*Data are median (minimum-maximum) in ppm on a wet weight basis. Shaded results are significantly higher (yellow) or lower (blue) than in other regions (Kruskal-Wallis with post-hoc Wilcoxon tests). Elements with >50% nondetectable results were excluded from analysis. See Supplementary Table S2 for all significant post hoc comparisons.

**Table 5:** Significant differences in concentrations of elements in Agassiz’s desert tortoises (*Gopherus agassizii*) based on primary disease at necropsy.*

Tissue	Element	*N*	Trauma	*N*	Cutaneous dyskeratosis	*N*	Infection/inflammation	*N*	Malnutrition	*N*	Mycoplasmosis	*N*	Urolithiasis	*P* value	Test statistic *(H)*
Keratin	Se	12	0.2 (0.1–0.3)	7	1.0 (0.2–2.4) ↑	6	0.2 (0.1–1.8)	5	0.2 (0.2–0.3)	5	0.2 (0.1–1.0)	2	0.3 (0.2–0.3)	0.022	12.0
	Zn	12	44 (23–78)	7	37 (28–67)	6	29 (19–44)	5	49 (30–84)	5	47 (37–55)	2	17 (16–18)	0.044	11.4
Kidney	Ca	13	310 (46–1,650) ↑	7	105 (75–290)	8	118 (110–320)	2	510 (120–900)	5	210 (82–260)	2	870 (440–1,300)	0.022	13.2
	Cd	13	0.4 (0.2–3.0) ↓	7	1.7 (0.9–3.9)	8	1.0 (0.5–5.2)	5	0.7 (0.3–1.2)	5	1.8 (1.3–5.2)	2	3.3 (1.3–5.3)	0.017	13.9
	Fe	13	78 (20–220) ↑	7	23 (14–66)	8	20 (14–110)	2	29 (19–38)	5	22 (17–29)	2	58 (39–78)	0.012	14.7
	Zn	13	19 (13–51)	7	31 (16–34)	8	26 (22–71)	5	130 (22–230) ↑	5	30 (21–41)	2	22 (17–26)	0.025	12.8
Liver	Ca	14	150 (45–1800) ↑	9	78 (26–90)	8	57 (21–240)	4	390 (52–870)	5	78 (56–180)	2	655 (530–780)	<0.01	15.2
	Cd	14	0.2 (0.1–1.1) ↓	9	0.6 (0.1–0.9)	8	0.2 (0.2–0.5)	7	0.5 (0.2–1.2)	5	1.2 (0.2–1.8)	2	1.6 (0.9–2.3)	0.005	16.6
	Cu	14	4.1 (1.1–10.0) ↓	9	12 (2.5–40.0)	8	2.9 (0.9–8.7) ↓	7	12.0 (4.9–75.0)	5	9.0 (1.0–30.0)	2	16.0 (12.0–20.0)	0.007	15.8
	Fe	14	278 (74–620) ↓	9	850 (190–1,200)	8	493 (250–1,300)	7	590 (470–1800)	5	1,650 (270–3,300)	2	1,600 (1400–1800)	0.001	20.5
	Na	14	1,200 (620–1900)	9	1,100 (640–2000)	8	908 (390–1800)	5	2,100 (880–3,700)↑	5	840 (470–2,100)	2	2,300 (1800–2,800)	0.043	11.4
	Se	14	0.2 (0.1–0.5) ↓	9	0.7 (0.2–2.1)	7	0.4 (0.1–3.5)	2	0.6 (0.3–0.9)	5	0.6 (0.3–1.5)	2	0.6 (0.5–0.7)	0.017	13.8
	Zn	14	20 (10–51) ↓	9	35 (15–62)	8	22 (9–62)	6	45 (21–98)	5	37 (9–52)	2	87 (33–140)	0.027	12.6

*Data are median (minimum-maximum) in ppm on a wet weight basis. Shaded results are significantly higher (yellow) or lower (blue) than those in other disease groups (Kruskal-Wallis with post-hoc Wilcoxon tests). Elements with >50% nondetectable results were excluded from analysis. See Supplementary Table S2 for all significant post hoc comparisons.

**Table 6:** Significant differences in concentrations of elements between reference and diseased Agassiz’s desert tortoises (*Gopherus agassizii*) in the Mojave and Colorado (western Sonoran) deserts of California, United States.

Tissue	Element	Trauma		Diseased		*P* value	Test statistic (*S*)
		No.	Median (minimum-maximum)	No.	Median (minimum-maximum)		
Kidney	Ca	13	310 (46–1,650)	24	118 (75–01300)	0.039	313
	Cd	13	0.4 (0.2–3.0)	27	1.3 (0.3–5.3)	0.015	182
	Cu	13	1.7 (0.1–6.6)	24	2.3 (0.1–13.0)	0.029	178
	Fe	13	78 (20–220)	24	23 (14–110)	<0.001	357
	K	11	1,600 (995–2,460)	15	2,100 (1470–3,000)	0.046	110
	Zn	13	19 (13–51)	27	28 (16–230)	0.005	168
Liver	Cd	14	0.2 (0.1–1.1)	31	0.5 (0.1–2.3)	0.004	203
	Cu	14	4.1 (1.1–10.0)	31	8.4 (0.9–75.0)	0.022	228
	Fe	14	278 (74–620)	31	850 (1990–3,300)	<0.001	160
	Hg	14	0.12 (0.05–0.35)	25	0.28 (0.04–0.82)	0.008	189
	K	10	1,510 (1170–2,350)	11	2,240 (1370–2,680)	0.014	75
	P	14	1750 (890–4,000)	29	2,300 (900–3,700)	0.033	226
	Se	14	0.2 (0.1–0.5)	24	0.6 (0.1–3.5)	<0.001	158
	Zn	14	20 (10–51)	30	34 (9–140)	0.008	209

*Data are median (minimum–maximum) in ppm on a wet weight basis. Shaded results are significantly higher (yellow) or lower (blue) than reference tortoises (Wilcoxon 2-sample test). No significant differences were found in keratin results between trauma and diseased tortoises. Elements with >50% nondetectable results were excluded from analysis.

**Figure 3:**
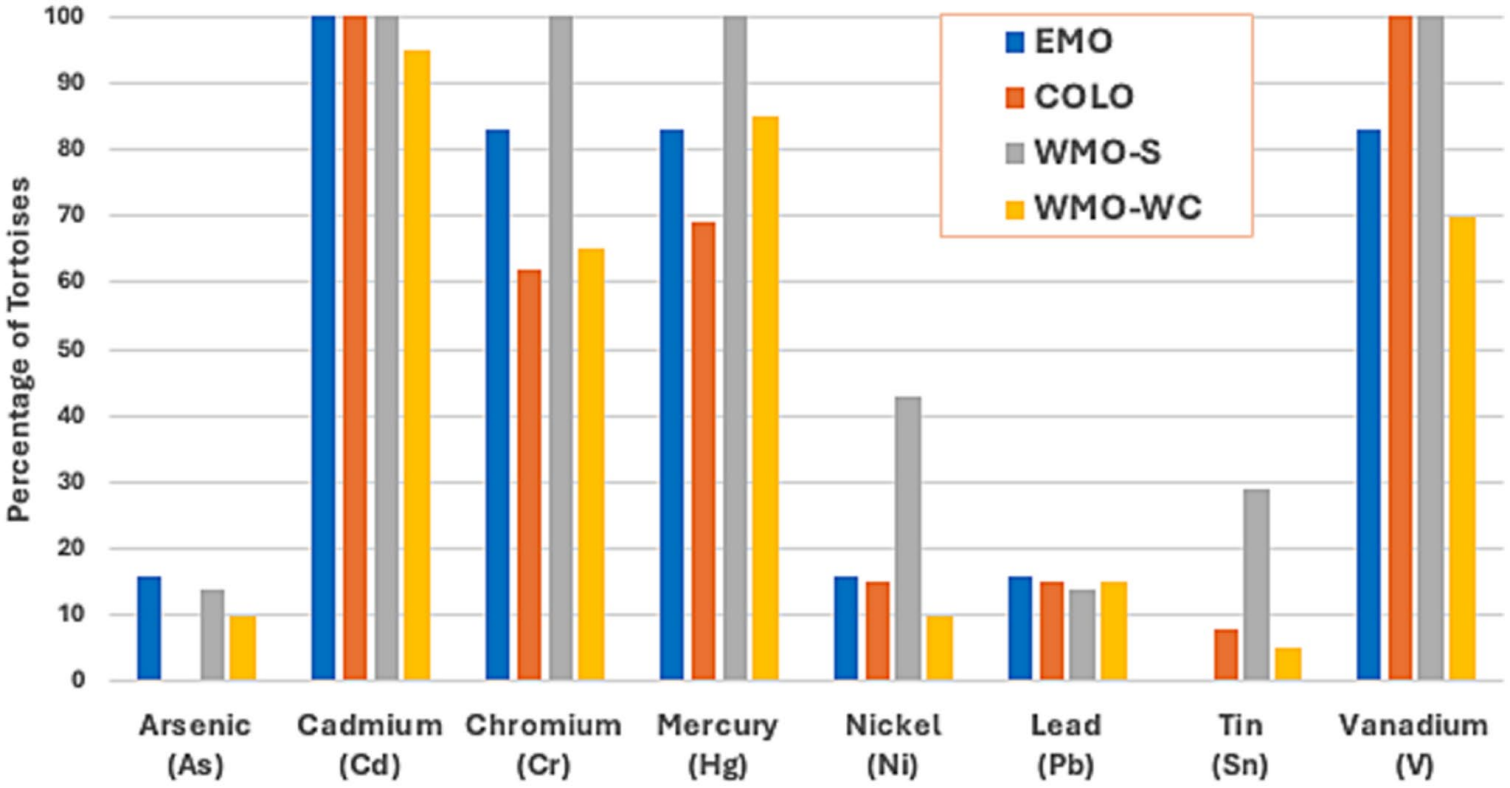
Prevalence of toxic metals in 46 Agassiz’s desert tortoises (*Gopherus agassizii*) salvaged from 4 regions of the Mojave and Colorado (western Sonoran) deserts in California, United States. As and Ni were found only in adults, while Pb and Sn occurred primarily in juveniles. A similar proportion of juvenile and adults had detectable levels of Cd and Cr, while a lower proportion of juveniles had detectable levels of Hg and V. EMO, East Mojave Desert; COLO, Colorado Desert; WMO-S, West Mojave Desert-southern section; WMO-WC, West Mojave Desert-western and central sections.

The prevalence of any detectable values for the 8 potentially toxic metals (As, Cd, Cr, Hg, Ni, Pb, Sn, and V) was 60–100% for Cd, Cr, Hg, and V, with Cr and Hg less prevalent in the EMO and COLO regions (Figure 3). Arsenic was not detected in tortoises in the COLO region. Ni and Sn were found in a higher proportion of tortoises in the WMO-S region (Supplementary Table S3).

“High” outlying values, defined as rare detectable values for heavy metals with ≥90% nondetectable results (As, Au, Ni, Pb, Sn) or as outliers by the >1.5 X IQR rule (other elements), were tabulated for adults and juveniles (Table 7; Supplementary Table S4). Most tortoises (22/35, 62.9%) had more than one high value. Five of 8 (62.5%) detectable Pb levels and 2/4 (50.0%) detectable Sn levels were in juveniles. The 2 adult tortoises with detectable Sn levels both had mycoplasmosis. Detectable As, Au, and Ni levels were found only in adults, and all 4 tortoises with detectable As values had infections. Twelve adult tortoises (8 diseased) had high Ni levels, and 5 of these had concurrent high values for other metals (As, Cd, Cr, Zn). The highest outlying Ni value (200 ppm) was found in scute keratin from an EMO (Fenner Valley) tortoise with cutaneous dyskeratosis. A higher proportion of tortoises with cutaneous dyskeratosis (*vs* other diseases) had high metal concentrations in keratin (6/21, 28.6%) compared with high kidney (3/16, 18.8%) or liver (1/21, 4.8%) concentrations. Of 5 Hg outliers, 2 were in kidney from adult tortoises in the EMO Fenner Valley region, and 2 were in keratin from tortoises that were killed by vehicles at Fort Irwin. Only 2 outlying low values were identified: 2 tortoises with trauma had low Na concentrations (880 and 870 ppm) in kidney tissue.

**Table 7:** Range of high outlying concentrations of trace minerals and heavy elements in keratin, kidney, and liver of Agassiz’s desert tortoises (*Gopherus agassizii*) from the Mojave and Colorado deserts of California, United States, between 1993 and 2000.*

Element (unit)	*n*	Keratin	*n*	Kidney	*n*	Liver	Total (*n*)
Al (ppm)	1	272	4	16–204	2	66–370	7
As (ppm)	2	11–15	1	1.9	1	1.9	4
Au (ppm)	3	0.3–5.1	2	1.8–2.0	3	0.6–1.8	8
B (ppm)	1	15.4	0	—	0	—	1
Ba (ppm)	3	6.1–7.9	5	1.1–3.7	6	1.0–7.8	14
Ca (ppm)	2	4,300–5,900	5	900–1,650	6	450–1,600	13
Cd (ppm)	1	3.5	3	5.2–5.3	2	1.8–2.3	6
Co (ppm)	0	—	1	1.2	0	—	1
Cr (ppm)	2	0.8–1.3	1	0.7	2	3.2–7.1	5
Cu (ppm)	0	—	3	6.6–13	5	30–75	8
Fe (ppm)	1	350	1	220	2	3,000–3,300	4
Hg (ppm)	3	0.19–0.50	2	0.76–1.15	0	—	5
K (ppm)	4	617–4,803	0	—	0	—	3†
Mg (ppm)	2	230–250	3	280–470	4	280–370	9
Mn (ppm)	1	7.8	3	3.2–8.3	3	2.8–15.0	7
Mo (ppm)	0	—	1	3.6	2	5.0–5.1	3
Na (ppm)	2	1,100–1700	1	4,700	1	3,700	4†
Ni (ppm)	4	2.1–200	2	0.9–7.5	6	0.7–3.0	12
P (ppm)	1	2000	0	—	1	4,000	2
Pb (ppm)	3	1.5–2.6	2	0.7–3.8	3	0.5–2.1	8†
Se (ppm)	6	1.0–2.4	1	2.0	5	1.5–3.5	12
Sn (ppm)	3	0.6–9.0	0	—	1	0.2	4†
V (ppm)	0	—	3	0.9–2.4	4	2.0–5.1	7
Zn (ppm)	1	78	6	51–230	3	86–140	10

*Many tortoises had outliers in more than one tissue (see Supplementary Table S4 for individual tortoise data). † ≥ 50% of outliers were in juvenile tortoises.

All but 2 tortoises had outlying (high) values for one or more elements (Supplementary Table S4). Eleven (27.5%) tortoises had a single outlying value, 19 (47.5%) had 2–3 outliers, and 10 (25.0%) had 4 or more outliers. High Se outliers were found in 3 adult tortoises from the EMO Fenner Valley region, and 2 adults and 1 juvenile tortoise in the COLO region. High levels of Al and Mn were found in 2 tortoises that died of trauma at Fort Irwin in the WMO-WC region. Two tortoises with high values for liver Fe had mycoplasmosis. Three tortoises had >4 outliers affecting multiple tissues. One tortoise in the Sand Hills area of the WMO-S region (with mycoplasmosis) had high values for Al, Cd, Cu, Mo, Pb, Se, and V as well as detectable levels of Cr and Hg. Another tortoise, also in Sand Hills and with mycoplasmosis, had high As, Cd, Mo, Ni, and V as well as detectable levels of Cr and Hg. One tortoise killed by vehicular trauma in the WMO-WC region (Fort Irwin), had high Cr, Cu, Hg, Mn, Ni, and Zn as well as detectable levels of Cd, in multiple tissues. Seven tortoises had 4 outliers each.

## 4 Discussion

In this study, we critically assessed and consolidated the results of analysis of 27 elements in the tissues of 46 tortoises in the Mojave and Colorado deserts and interpreted them in the context of two size classes, geographic location, and primary disease. We found significant differences in elemental concentration by tissue, especially between keratin and internal organs (kidney and liver). We also found significant differences between adult and juvenile tortoises, tortoises from different desert regions, and in different disease conditions. Higher Se concentrations occurred in scute keratin from tortoises with cutaneous dyskeratosis. Importantly, we documented the presence of multiple potentially toxic elements in most tortoises. Potential adverse health effects in reptiles caused by metals of priority concern (Al, As, Cd, Cr, Cu, Hg, Mn, Ni, and Pb) include cancer, reproductive and developmental disorders, disruption of endocrine and immune functions, renal and hepatic dysfunctions, neurotoxic disorders, and genetic damage [(52, 59, 60), Table 12.7]. The United States Agency for Toxic Substances and Disease Registry produced a National Priority List^4^ for elements and organic compounds. Of the trace elements in our study, only Au, Fe, Sn, and Tl were missing from the National Priority List, although these elements can be toxic to humans, depending on dosage, sex, and other characteristics.

Under ideal circumstances, we would know or be able to estimate the toxicity of the elements to desert tortoises and some other testudines. Such data are unavailable and unlikely to become so because of the federal and state listings of these species as threatened and endangered [e.g., (1, 6)]. In the present study diseased tortoises had significantly lower Ca and Fe and higher Cd, Cu, and Zn in kidneys, and higher Cd, Cu, Fe, Hg, Se, and Zn in livers compared with tortoises dying of trauma. Like the desert tortoises in this study, multiple potentially toxic elements have also been reported in scute, kidney, and liver of sea turtles found dead, dying, or stranded (61–66). Compared with sea turtles, scutes of desert tortoises had higher concentrations of Al, As, Cd, Ni, and Pb (Supplementary Table S5). Similarly, Al, Ba, Co, Fe, Hg, Mn, V, and Zn were higher in kidneys, and Al, Ba, Cu, Fe, Hg, Mn, V, and Zn were higher in livers of desert tortoises. These comparisons with sea turtles, however, are based on comparatively few samples such that direct comparison and interpretation are difficult. In addition, the sea turtles live in the marine environment, travel widely, and diets range from herbivory to carnivory. Analytical methods and handling of nondetectable results also may have differed among studies. The presence of multiple, potentially toxic elements in sea turtles and desert tortoises presents an additional challenge: which element may have contributed to illness or stranding? Frossard et al. (59) suggested that effects of multiple elements cannot be separated, and provided evidence for genotoxic effects of multi-element exposure in hatchling turtles from multiple sources: maternal, eggshell, and nest. Tchounwou et al. (67), in a study of high priority toxic elements in humans, noted that chronic low dose exposure to multiple elements was a major public health concern. Our observations indicated that potentially toxic elements, whether singly or in multiples, were associated with diseases in the desert tortoise.

Reptiles with great longevity have the potential to accumulate heavy metals and other elemental toxicants over time when living in contaminated areas. Bioaccumulation is a another challenging and potentially complex topic with multiple factors: location, diet, trophic position, size, sex, size, and health. For desert tortoises, we have small sample sizes of juveniles and adults from different locations and regions. We divided tortoises into juvenile and adult classes by size; we did not separate adults further by relative age (young, middle-aged, old) or size. The complexity of age in bioaccumulation is exemplified by a long-term, mark-recapture study of Hg in American alligators, *Alligator mississippiensis* (68). The accumulation of Hg peaked at an age of 30 to 40 years and was lower in young and old individuals. In another study, size was important in the lizard, *Psammodromus algirus*; heavy metals increased with size of the lizard (69).

### 4.1 Tortoises with trauma

Designation of a “trauma” group of tortoises killed primarily by vehicles and without apparent underlying diseases enabled comparisons with diseased tortoises having well-documented metabolic and inflammatory conditions. While disease cannot be completely ruled out in all tortoises with trauma, evidence of disease at necropsy was lacking or minimal in comparison with diseased tortoises. Also, while undetected underlying disease could affect tortoise susceptibility to predation, it is unlikely to contribute to collisions with vehicles. The predominance of tortoises killed by trauma in the WMO-WC region likely explains lower values for several element concentrations in kidney and liver. Most of these tortoises were from areas with degraded habitat, in the proximity of and on roads and on a military reservation where military vehicles, including tanks, engaged in war games (10). Although the trauma group had significantly lower concentrations of Cd, Hg and Zn, many individual tortoises with trauma had multiple detectable metals with toxic potential. In addition, Se levels were lower in tortoises with trauma vs. diseased tortoises, likely reflecting differences in the availability of dietary Se in soil and plants in the WMO-WC region.

### 4.2 Elements, physiology, and disease

Differences in macrominerals (Ca, P, Na, K, Mg) were more likely related to metabolic changes and/or dehydration associated with disease processes than to environmental contamination. The two low kidney Na values in tortoises with trauma suggested dilute urine secondary to drinking water. Calcium is a primary component of scute keratin, while other macrominerals were present in much lower concentrations. Differences in wet weights of kidney and liver could affect macromineral concentration, but only Ca differed significantly and the effect was small. Calcium concentration was significantly lower in diseased tortoises. This could, in part, be associated with decreased intake of Ca with malnutrition or hyporexia, and with hypoalbuminemia secondary to protein malnutrition (a large portion of Ca is albumin-bound). Tortoises with urolithiasis had higher Ca concentrations than tortoises with other diseases, likely because of decreased urinary excretion but also likely reflecting Ca urate crystal deposition in tissue, as was found in one tortoise.

Se concentrations differed significantly based on tissue, region, and disease, with lower concentrations in keratin (in all tortoises combined), and higher concentrations in tortoises in the EMO region and with cutaneous dyskeratosis. Se was proposed as a potential cause or contributor to this disease (49), and our results support this hypothesis. Elevated levels of Se (selenosis) are linked to dyskeratosis of keratin in hoofed animals (70), with histologic findings that resemble those in affected tortoises (71). Selenosis results from chronic ingestion of seleniferous plants in areas of the western United States with alkaline soils. Notably, tortoises with cutaneous dyskeratosis had increased Se primarily in keratin and liver, tissues used as long-term indicators of Se status in domestic animals (70). This was despite some tortoises in the EMO region having low plasma Se (and vitamin E) levels (Supplementary Table S1). Plasma Se is bound to plasma proteins and prone to fluctuation such that keratin and liver are considered better samples for postmortem testing. In experimental treatments with different Se doses in the yellow-bellied slider turtle (*Trachemys scripta scripta*), the most notable results at higher doses (30 mg/kg for 5 weeks) were elevated mortality rates, accumulation in kidney, liver, muscle, and blood, and changes in claws (72, 73). The more severe lesions in the claws included epidermal necrosis and separation of the cornified layer from the dermis.

Cu and Fe values were significantly higher in liver than in other tissues, and both elements were significantly higher in the liver of diseased tortoises. Cu and Fe are essential elements that form part of many enzymes, proteins, and cofactors in the liver (74). The higher concentration of Fe in diseased tortoises was consistent with sequestration of Fe in macrophages due to cytokine activation in inflammatory (e.g., mycoplasmosis) and other chronic diseases, as also occurs in other species (40). The two tortoises with high outlying Fe values in the present study both had mycoplasmosis, and hemosiderosis was noted in the liver (and spleen) of these and other ill tortoises at necropsy.

Hg also was higher in liver, especially in diseased tortoises, consistent with earlier observations by Jacobson et al. (40) in tortoises with mycoplasmosis. Liver is a primary site for Hg accumulation [e.g., (66, 75)], which has a high potential for toxicity. Fewer juveniles had detectable Hg concentrations, suggesting a slow, chronic accumulation of Hg that primarily affects adults.

A high proportion of results were nondetectable for some analytes, especially Ag, As, Au, Ni, Pb, Sn, and Tl. These elements are likely nonessential (52), with none expected in the body normally, such that any detectable result was considered an outlier. Although statistical analyses should be interpreted with caution, measurable levels of nonessential elements could be cause for concern.

### 4.3 Potential sources of elements and contamination of the environment

Mines and mine tailings are a potential source of toxic elements (76, 77). Two tortoises from the Rand Mining District (WMO-WC region) had the highest outlying keratin values for Al (272 ppm) and As (15 ppm). Both tortoises also had measurable levels of Cd, Cr, Hg, Mn, Mo, and V and were diagnosed with severe inflammation. The tortoise with high As levels was salvaged from an active gold mine that used cyanide-heap leach processing. The Rand Mining District and surrounding lands are high in Hg, As, and Cr (14). Arsenic is considered a threat to human health (78, 79). Because of high levels of toxic wastes from historic mining in the Rand District, the USDI Bureau of Land Management was involved in cleaning up the site ~20 years after the tortoise was collected.^5^

Historic and recent military activities have left legacies of toxic metals, metalloids, and organic wastes in the soils (55, 77, 80), including As, Cd, Cr, Cu, Ni, Pb, Sb, V, and Zn. Fourteen of 20 tortoises in the WMO-WC region were from the National Training Center, Fort Irwin, an active military base used for force-on-force training with armored vehicles. Seven tortoises died of trauma and 5 were in a protected area of the Fort in head-start programs to raise juvenile tortoises for research on behaviors and survival [e.g., (81)]. One or more tissues of all tortoises had 2 to 7 elements with high outliervalues. An additional tortoise with 10 high outlier values of elements was on a military facility in WMO-S, the Marine Corps Air Ground Combat Center.

Fifteen of 17 tortoises (88.2%) from the EMO and COLO regions lived in historic military training areas (2 additional tortoises were outside military use areas) (82). Tortoises living here had 1 to 4 outlying high concentrations of trace minerals and heavy metals. The earliest training occurred in all but the Ivanpah Valley. From 800,000 to 1,000,000 soldiers under General George S. Patton were trained in tank warfare for World War II between 1942 and 1944 (10, 82). Several formal camps, 13 ranges for small caliber arms and mortar fire, and roads were developed. Tank tracks, exploded and unexploded ordnance, and decaying batteries provide lasting evidence of the exercises. The effects of military maneuvers in the EMO and COLO were evident on soils and plants (83–85). In the southern COLO, practice bombs were evident, likely from the adjacent Chocolate Mountains Aerial Gunnery Range. The second historic military action, named Desert Strike, occurred in 1964 in Ivanpah Valley, California, and parts of Nevada, and Arizona.^6^ The 2-week activity involved 100,000 personnel, >900 aircraft, and 500 tanks. No clean-up has occurred for either military operation.

Although tortoises have behavioral, physiological, and reproductive adaptations for living in deserts, anthropogenic activities have led to serious declines in populations (1–3, 86). Multiple potential elemental toxicants (e.g., Hg, Pb, and Se) in tissues of tortoises with different diseases also may be contributing to their decline. The warming climate with predictions of increased and prolonged droughts and rising temperatures is likely to further stress the physiology and behaviors of tortoises, requiring more time spent in burrows, construction of deeper burrows to avoid heat stress in summer, drier soils, and less predictability of food and water (87–91). Droughts, and the 1999–2020 megadrought and heat waves affected primary productivity, the shrubs tortoises use for cover, and availability of annual forage (e.g. (22, 26)). Most declining tortoise populations in critical habitat units experienced additional losses between 2004 and 2014 on a landscape scale, to a point of non-viability (2, 86). With continued warming, deaths of tortoises from starvation and dehydration are likely to increase (22, 32, 92), and with dehydration, potentially toxic elements may become more concentrated in tissues. This may be exacerbated when dehydration is accompanied by malnutrition, starvation, and emaciation (32).

The present study has several limitations. While the data enabled associations to be made between potentially toxic elements and disease, we did not determine causality, which would require experimental studies that are infeasible in desert tortoises because of their federal threatened and state endangered status (1, 6). Many of the tortoises had multiple concurrent disease processes, making identification of the primary disease difficult in some cases. For example, several tortoises with cutaneous dyskeratorsis had loss of body condition, similar to (although usually less severe than) tortoises with primary malnutrition. For this reason, we compared elements in all diseased tortoises vs. tortoises with trauma in addition to comparing specific diseases. Another limitation of the study was the non-random and heterogeneous nature of the sampled population and the small (and variable) sample sizes, especially for some groups (e.g., juveniles, urolithiasis). Because of its exploratory nature, we chose to report *p* values of <0.05, recognizing that the likelihood of Type I errors (false positives) would be higher. Nonetheless, we have emphasized those findings with the most statistical support, and believe that inclusion of all of the results and disease findings could facilitate the generation of new hypotheses for future studies. In general, challenges exist with experimental studies on potential toxicants of rare, threatened, and endangered species (54). Such species are likely to have various elemental and organic pollutants from the environments in which they live. The synergistic effects of the pollutants/contaminants on the health of the animals have not been determined.

For the desert tortoise, we are unaware of experimental studies on the potential toxicity, lethality, or contribution to diseases of elements, although Hg may have been a contributing factor in tortoises with mycoplasmosis at the Desert Tortoise Research Natural Area (40). Additionally, we are unaware of research on maternal transfer of toxic elements to eggs, or for eggs to absorb trace elements in the nest, although this has been documented for other species of reptiles [e.g., (52, 59)]. Minimal sampling of blood, eggs, eggshells, soils used for nesting, and possibly limited samples of shell are potential options to limit invasive sampling in future investigations on the role of toxic elements in this free-ranging species. Blood samples also can provide evidence of genetic damage and oxidative stress [e.g., (55)]. However, elements occurring in peripheral blood do not provide information on accumulation in tissues such as kidney and liver. The coupling of health evaluations with collection of data on toxicants provides substantial advantages to understanding the potential effects of toxicants, e.g., Miguel et al. (63). In the present study, the salvage of ill and dying tortoises provided the unique opportunity to obtain complete necropsies coupled with evaluation of elemental toxicants in tissues.

In conclusion, desert tortoises live in circumscribed areas in and on the ground in close contact with soil; they eat plants, rocks, and soil; and breathe air and dust within a few centimeters of the ground. Thus, metals and other elements found in keratin, kidneys, and liver reflect local, anthropogenically disturbed environments, and in the case of Se may reflect natural soil differences. Importantly, diseased tortoises have higher levels of trace elements than tortoises with trauma and no apparent underlying disease, including elements known to be toxic or nonessential to life. Prospective analysis of representative tortoise populations using noninvasive sampling (e.g., of blood or scutes) would be useful for further assessing the role of Se and other potentially toxic elements in disease pathogenesis. The long-lived tortoise can join other reptiles—crocodilians, sea turtles, and aquatic turtles—as sentinels and bio-monitors of contaminants in the environment (52, 53).

## Data Availability

The raw data supporting the conclusions of this article will be made available by the authors, without undue reservation.

## Footnotes

^1^https://cal.span.org/meeting/cfg_20240418

^2^www.nist.gov

^3^https://www.aihaaccreditedlabs.org/

^4^https://www.atsdr.cdc.gov/spl/index.html

^5^https://www.blm.gov/programs/aml-environmental-cleanup/aml/state-information/california/rand-historic-mining-complex

^6^www.HMdb.org
