# Supplementary material for: Potentially toxic elements in wild Agassiz’s desert tortoises: tissue concentrations and association with disease
Source: Front Vet Sci. 2024 Nov 21;11:1481367. doi: 10.3389/fvets.2024.1481367 (PMC11617541; doi:10.3389/fvets.2024.1481367)
Supplement: Supplementary file 1 [file Table_1.docx]

**Supplementary Table 1.** Summary of the 46 necropsied Agassiz’s desert tortoises (*Gopherus agassizii*) salvaged in the Mojave and Colorado deserts of California, USA.* WMO-W = Western Mojave Desert region, WMO=C = Central Mojave Desert region, WMO-S = Southern Mojave Desert region, NEMO = Northeastern Mojave Desert region, ECO = eastern Mojave Desert region, NCO = northern Colorado Desert region, and ECO = Eastern Colorado Desert region.

| **ID** | **Age/Sex** | **MCL (mm)** | **BW (kg)** | **Date** | **Location in California, USA** | **Clinical, Laboratory and Necropsy Findings*** | **Region** | **Primary Disease Process** |
| --- | --- | --- | --- | --- | --- | --- | --- | --- |
| 6 | Adult/ Female | 191 | 1.15 | 4 -Jun-93 | Highway 58, Kramer Junction, San Bernadino County | Clinical: movements limited to near major highway; lethargic, flaky shell, entombed. Lab: mild anemia, hypoprotenemia; *Mycoplasma* negative. Necropsy: multifocal flaking shell lesions, epidermal pustules with aggregates of necrotic heterophils, dermal heterophils and fungal hyphae (also in epidermis of shell), heterophilic infiltrates in multiple internal organs, gelatinous fat. Diagnosis: severe cutaneous fungal infection with septicemia. | WMO-W | Infection/inflammation |
| 8 | Juvenile/ Male | 126 | 0.47 | 16-Jun-93 | Chuckwalla Bench, Riverside County | Clinical: shell lesions, missing scutes. Lab: high bilirubin and ALP; *Mycoplasma* negative. Necropsy: white discolored shell lesions with cracks and clefts, epithelial atrophy and dermal irregularity, severely affected bones with osteoclastic resorption. Diagnosis: severe cutaneous dyskeratosis. | ECO | Cutaneous dyskeratosis |
| 9 | Adult/ Male | 284 | 4.03 | 16-Jun-93 | Chuckwalla Bench, Riverside County | Clinical: light to moderate shell lesions on anterior surface of gular horn. Lab: *Mycoplasma* negative. Necropsy: several scute defects, scratches, grooves; minimal shell lesions microscopically (occasional cracks and fissures). Diagnosis: cutaneous dyskeratosis. | ECO | Cutaneous dyskeratosis |
| 10 | Adult/ Female | 227 | 1.90 | 16-Jun-93 | Chuckwalla Bench, Riverside County | Clinical: severe shell lesions on plastron. Lab: *Mycoplasma* negative. Necropsy: white flaky shell lesions with microscopic cracks and fissures, clefts, altered stratum corneum, dermal plates, irregular bony plates, osteoclastic resorption. Diagnosis: cutaneous dyskeratosis. | ECO | Cutaneous dyskeratosis |
| 12 | Adult/ Male | 286 | 4.25 | 12-Aug-93 | Hackberry Mountains, San Bernadino County | Clinical: severe burn marks on shell, melted shell, burns on legs and nose, toenails burnt off. Lab: anemia, leukopenia, hypoalbuminemia, hypocholesterolemia, increased ALT/AST; *Mycoplasma* serology equivocal (interpreted as negative). Necropsy: discolored skin with single leg ulcer with bacteria, blackened subcutis with exudate, multifocal transmural necrotizing inflammation of small and large intestine, serosal inflammation of lung and kidney, renal tubular degeneration, granulocytic depletion in marrow. Diagnosis: inflammation and septicemia secondary to necrotizing enteritis and severe burn injury. | EMO | Infection/inflammation |
| 15 | Adult/ Female | 176 | 0.74 | 27-Apr-94 | Highway 58, Kramer Junction, San Bernadino County | Hit by car. No laboratory testing. Necropsy: shell fractured, liver lacerations, mild acute pneumonia (likely secondary to fractured shell), minor skin infection (with bacteria, fungi). Mild hepatic and splenic hemosiderosis. Diagnosis: blunt trauma. | WMO-W | Control (trauma) |
| 16 | Juvenile/ Male | 52 | 0.3 | 4-May-94 | Ivanpah Valley, East San Bernadino County | Clinical: slow growth, shell lesions with discoloration and peeling laminae. Lab: mild anemia; *Mycoplasma* negative. Necropsy: shell lesions consistent with mild multifocal cutaneous dyskeratosis; moderate to marked osteopenia (consistent with malnutrition); hepatic hemosiderosis. Diagnosis: 1) malnutrition, 2) cutaneous dyskeratosis. | NEMO | Malnutrition  Other: cutaneous dyskeratosis |
| 17 | Adult/ Female | 230 | 2.5 | 4-May-94 | Johnson Valley, West San Bernadino County | Clinical: robust condition, peeling laminae on plastron. Lab: high bile acids; *Mycoplasma* negative. Necropsy: focal scute defects, unique shell lesions with extensive exudation within keratin layer (consistent with bacterial etiology, intralesional fungi isolated), bone thin and necrotic. Diagnosis: 1) bacterial and fungal necrotizing epidermitis and bone necrosis of shell, 2) focal cutaneous dyskeratosis. | WMO-S | Infection/inflammation Other: cutaneous dyskeratosis |
| 18 | Adult/ Female | 240 | 1.89 | 9-May-94 | Panorama Heights, North Joshua Tree National Monument, San Bernadino County | Clinical: severely emaciated, sunken flesh. Lab: anemia, leukocytosis, heterophilia, lymphocytosis, monocytosis, azotemia, hyperosmolality, hypophosphatemia, hyperkalemia; *Mycoplasma* culture positive. Necropsy: embedded cactus spines, mild inflammation of pharynx, nasal cavity, and lungs; skin pustules with bacteria and fungal hyphae; hepatic hemosiderosis. Diagnosis: 1) mycoplasmosis with associated inflammation, 2) dehydration and emaciation. | WMO-S | Mycoplasmosis  Other: dehydration and emaciation |
| 19 | Adult/ Female | 252 | 2.3 | 24-Oct-94 | Stoddard Valley, San Bernadino County | Clinical: abnormal behavior, weak, signs of upper respiratory tract disease. Lab: mild anemia, leukocytosis, heterophilia, monocytosis, eosinophilia; *Mycoplasma* serology positive. Necropsy: chronic moderate rhinitis. Diagnosis: 1) mycoplasmosis with associated inflammation. | WMO-S | Mycoplasmosis |
| 20 | Adult/ Male | 222 | 1.78 | 29-Nov-94 | Barstow, San Bernadino County | Clinical: emaciated, lethargic, abnormal behavior, nasal discharge, chipped scutes. Lab: marked anemia, panhypoproteinemia, azotemia, hyperosmolality, high CK; *Mycoplasma* serology positive. Necropsy: mild chronic rhinitis; mild to moderate interstitial pneumonia; hepatic, testicular, thyroid and skeletal muscle atrophy; hepatic hemosiderosis; focal fungal colonies on skin. Diagnosis: 1) mycoplasmosis, 2) dehydration and emaciation. | WMO-S | Mycoplasmosis  Other: dehydration and emaciation |
| 21 | Juvenile/ Female | 48 | 0.14 | 20-Dec-94 | Fort Irwin, San Bernadino County | Found dead, preserved by cold; dehydration and malnutrition in other tortoises at site. Sent frozen. No laboratory testing. Necropsy: marked autolysis so limited microscopic findings; bacterial colonization in multiple organs; severe osteopenia of shell and leg bones; skeletal muscle atrophy; hepatic hemosiderosis. Diagnosis: 1) shell osteopenia and muscle atrophy consistent with malnutrition, 2) bacterial septicemia. | WMO-C | Malnutrition  Other: infection/ inflammation |
| 22 | Juvenile/ Female | 65 | 0.53 | 20-Dec-94 | Fort Irwin, San Bernadino County | Found dead, preserved by cold; dehydration and malnutrition in other tortoises at site. Sent frozen. No laboratory testing. Necropsy: soft pliable shell; severe osteopenia in shell and bones, hepatic and skeletal muscle atrophy, mild nasal inflammation. Diagnosis: shell osteopenia and muscle atrophy consistent with malnutrition. | WMO-C | Malnutrition |
| 23 | Adult/ Male | 265 | 1.68 | 21-Mar-95 | Fort Irwin, San Bernadino County | Clinical: weight loss, weak, lethargic. Lab: anemia, marked azotemia and hyperosmolality, hyperuricemia, hyperphosphatemia, hyperbilirubinemia, high bile acids, cholesterol, and CK; concentrated urine; *Mycoplasma* serology equivocal (interpreted as negative). Necropsy: Large urolith in bladder, moderate to marked renal tubular necrosis with intralesional urate crystals; hepatic hemosiderosis; skeletal muscle degeneration; hepatic and pancreatic atrophy; mild colitis. Diagnosis: 1) urolithiasis, 2) severe dehydration 3) renal and articular gout. | WMO-C | Urolithiasis  Other: dehydration and renal disease (gout) |
| 24 | Adult/ Male | 285 | 2.07 | 21-Mar-95 | Ivanpah Valley, East San Bernadino County | Found dead in burrow, severe decomposition. No laboratory testing. Necropsy: marked autolysis; focally extensive lesions of cutaneous dyskeratosis, shell flaking at seams; focal gastritis with intralesional thorn and bacteria; mild to marked heterophilic enteritis; heterophilic infiltrate in lungs; urolith, moderate cystitis. Diagnosis: 1) urolithiasis, 2) cutaneous dyskeratosis, 3) gastrointestinal inflammation. | NEMO | Urolithiasis  Other: cutaneous dyskeratosis, infection/inflammation |
| 25 | Juvenile/ Unknown | 70 | 0.43 | 21-Mar-95 | Fort Irwin, San Bernadino County | Found dead and covered with dirt; had been dehydrated, lethargic, weight loss. Sent frozen. No laboratory testing. Necropsy: marked autolysis of internal organs; fragile and fractured scutes, carapace, and plastron; muscle mass reduced by ~50%, no coelomic fat, prominent ribs; mild to moderate osteopenia of shell and bones. Diagnosis: long-term malnutrition. | WMO-C | Malnutrition |
| 26 | Juvenile/ Unknown | 53 | 0.26 | 21-Mar-95 | Fort Irwin, San Bernadino County | Found dead, covered with ants, very dry year with little if any food. Sent frozen. No laboratory testing. Necropsy: marked autolysis; eyes sunken, ribs prominent, no coelomic fat, muscle mass reduced ~50%, mild to moderate osteopenia of shell and bones. Diagnosis: long-term malnutrition. | WMO-C | Malnutrition |
| 27 | Adult/ Male | 178 | 0.90 | 1-May-95 | North Ward Valley, San Bernadino County | Hit by car. Sent frozen. No laboratory testing. Necropsy: moderate autolysis; fracture in plastron and in scutes/seams; viscera protruding from shell defect; dislocated leg; ruptured spleen; moderate skeletal muscle degeneration; focal tongue granuloma with thorn. Diagnosis: 1) blunt trauma. | NCO | Control (trauma) |
| 28 | Adult/ Female | 215 | 1.65 | 1-May-95 | Upper Ward Valley, San Bernadino County | Clinical: wet eyes, swollen eyelids, shelllesions on plastron, carapace, forelegs. Lab: anemia, leukocytosis, monocytosis, basophilia, hyperbilirubinemia, high bile acids; *Mycoplasma* negative. Necropsy: discolored plastron, scute defects with exposed bone and osteopenia, dermatitis; gelatinous body fat; heterophilic and lymphocytic infiltrates in liver, lung, spleen, brain, conjunctiva, intestine, et al. (consistent with generalized bacterial infection); hepatic atrophy. Diagnoses: 1) bacterial inflammation/septicemia; 2) cutaneous dyskeratosis. | NCO | Infection/inflammation  Other: cutaneous dyskeratosis |
| 29 | Adult/ Female | 245 | 3.17 | 25-May-95 | Central Pinto Basin, Joshua Tree National Park, Riverside County | Clinical: prior signs of upper respiratory tract disease. swollen eyelids, wet nostrils; motor oil droplets on carapace. Lab: high bile acids; *Mycoplasma* culture positive. Necropsy: mild nasal discharge, moderate to marked rhinitis, conjunctivitis, and proctitis; mild inflammation in multiple organs (e.g., spleen, larynx, myositis); hepatic atrophy. Diagnosis: mycoplasmosis with associated inflammation. | ECO | Mycoplasmosis |
| 30 | Adult/ Male | 250 | ND | 20-Jun-95 | Desert Tortoise Natural Area, Kern County | Found dead, severely decomposed, wedged in burrow. Sent frozen. No laboratory testing. Necropsy: severe autolysis so no histologic examination; large urolith found. Diagnosis: urolithiasis. | WMO-W | Urolithiasis |
| 31 | Adult/ Male | 271 | 3.73 | 18-Jul-95 | Rand Mountains, East Kern County | Clinical: lived in gold mine area, peeling laminae, signs of upper respiratory tract disease. Necropsy: scutes depressed centrally, flaking; heterophilic and lymphocytic infiltrates in chin glands, eyelids, and nasal cavity (with osteolysis); lymphocytosis, monocytosis, mild increase in CK, low vitamin A; *Mycoplasma* negative. Diagnosis: inflammation of head structures. | WMO-W | Infection/inflammation |
| 32 | Adult/ Female | 210 | 1.75 | 18-Jul-95 | Rand Mountains, East Kern County | Clinical: lived in gold mine area, advanced shell lesions typical of cutaneous dyskeratosis. Lab: leukocytosis, heterophilia, eosinophilia, basophilia, mild increase in AST, CK, low vitamin A; *Mycoplasma* negative. Necropsy: scute abnormalities, flaking carapace, skin shedding, with lymphocytic infiltrate and fungal colonization; minor heterophilic infiltrates in lung and larynx. Diagnosis: 1) multifocal necrotizing epidermitis with intralesional fungi; 2) moderate cutaneous dyskeratosis, | WMO-W | Infection/inflammation, Other: cutaneous dyskeratosis |
| 33 | Adult/ Female | 203 | 1.15 | 11-Jun-96 | Fort Irwin, San Bernadino County | Struck by military vehicle, found dead. No laboratory testing. Necropsy: lesions consistent with trauma. Diagnosis: trauma | WMO-C | Control (trauma) |
| 34 | Adult/ Male | 260 | 2.15 | 11-Jun-96 | Fort Irwin, San Bernadino County | Struck by military vehicle, found dead. No laboratory testing. Necropsy: lesions consistent with trauma. Diagnosis: trauma | WMO-C | Control (trauma) |
| 35 | Adult/ Male | 163 | 0.54 | 11-Jun-96 | Fort Irwin, San Bernadino County | Struck by military vehicle, found dead. No laboratory testing. Necropsy: lesions consistent with trauma. Diagnosis: trauma | WMO-C | Control (trauma) |
| 36 | Adult/ Male | 256 | 2.20 | 24-Sep-96 | Sheephole Mountains, San Bernadino County | Found overturned and dead, evidence of acute predation). No laboratory testing. Necropsy: autolysis precludes histologic examination. | NCO | Control (trauma) |
| 37 | Adult/ Male | 200 | 1.45 | 4-Mar-97 | Highway 58, Harper Lake Road, San Bernadino County | Hit by car, found dead. No laboratory testing. Necropsy: lesions consistent with trauma. Diagnosis: trauma | WMO-W | Control (trauma) |
| 38 | Adult/ Male | 262 | 2.70 | 1-Apr-97 | Chuckwalla Bench, Riverside County | Clinical: moderate to advanced shell lesions. Lab: low vitamin E and selenium. Necropsy findings and diagnosis: 1) cutaneous dyskeratosis with intralesional fungi and multicentric inflammation of shell; 2) mild hepatic, pancreatic and testicular atrophy (loss of condition); 3) multifocal degeneration of heart and skeletal muscle (suggest nutritional deficiency). | ECO | Cutaneous dyskeratosis  Other: infection/ inflammation, loss of condition/nutritional deficiency |
| 39 | Adult/ Female | 246 | 1.95 | 13-May-97 | Chuckwalla Bench, Riverside County | Found dead. No laboratory testing. Necropsy findings and diagnosis: 1) moderate atrophy of liver and acute skeletal muscle degeneration (suggest nutritional deficiency), 2) mild pneumonia. | ECO | Malnutrition |
| 40 | Adult/ Male | 202 | 1.60 | 28-May-97 | Chuckwalla Bench, Riverside County | Clinical: moderate to severe shell lesions. Lab: low vitamin E and selenium. Necropsy findings and diagnosis: 1) cutaneous dyskeratosis with intralesional fungi and fungal colonization of skin; 2) moderate liver and testicular atrophy (loss of condition). | ECO | Cutaneous dyskeratosis  Other: loss of condition/nutritional deficiency |
| 41 | Adult/ Male | 200 | 1.50 | 28-Aug-97 | Twenty-nine Palms, San Bernadino County | Clinical: severe shell lesions with loss of scute material, exposed dermal bone, flaking, amputated leg. Lab: anemia, leukocytosis, heterophilia, monocytosis, basophilia; hyperbilirubinemia, mild increase in ALP, mild hypoproteinemia, hypoalbuminemia; *Mycoplasma* negative. Necropsy: shell pustules, crusts, necrotic bone, and heterophilic inflammation with intralesional fungi (cultured *Chrysosporium*, *Alternaria*, *Epicoccum*, *Alternaria niger*); mild organ atrophy; hepatic and splenic hemosiderosis; mild skeletal muscle degeneration and liver atrophy. Diagnosis: 1) severe cutaneous fungal infection of shell, 2) multifocal cutaneous dyskeratosis | WMO-S | Infection/inflammation  Other: cutaneous dyskeratosis |
| 42 | Adult/ Male | 290 | 3.80 | 3-Oct-97 | Fort Irwin, San Bernadino County | Struck by military vehicle, found dead. No laboratory testing. Necropsy: lesions consistent with trauma, focal cutaneous dyskeratosis of plastron. Diagnosis: trauma. | WMO-C | Control (trauma) |
| 43 | Adult/ Female | 205 | 1.43 | 6-Feb-97 | Fort Irwin, San Bernadino County | Struck by military vehicle, found dead. No laboratory testing. Necropsy: lesions consistent with trauma; autolysis precluded histologic examination. Diagnosis: trauma. | WMO-C | Control (trauma) |
| 44 | Adult/ Male | 290 | 3.70 | 20-Apr-98 | Fort Irwin, San Bernadino County | Struck by military vehicle, found dead. No laboratory testing. Necropsy: lesions consistent with trauma, mild cutaneous dyskeratosis, mild hepatic degeneration. Diagnosis: trauma. | WMO-C | Control (trauma), |
| 45 | Adult/ Male | 220 | 2.10 | 16-Jun-98 | Fort Irwin, San Bernadino County | Struck by military vehicle, found dead. No laboratory testing. Necropsy: lesions consistent with trauma, mild-moderate cutaneous dyskeratosis at seams. Diagnosis: trauma. | WMO-C | Control (trauma) |
| 46 | Juvenile/ Female | 170 | 1.10 | 16-Jun-98 | Fort Irwin, San Bernadino County | Struck by military vehicle, found dead. No laboratory testing. Necropsy: lesions consistent with trauma. Diagnosis: trauma. | WMO-C | Control (trauma) |
| 47 | Adult/ Male | 230 | 2.30 | 16-Jun-98 | Fort Irwin, San Bernadino County | Struck by military vehicle, found dead. No laboratory testing. Necropsy: lesions consistent with trauma. Diagnosis: trauma. | WMO-C | Control (trauma) |
| 48 | Adult/ Male | 200 | 1.80 | 16-Sept-98 | Barstow, Highway 247, San Bernadino County | Hit by car, found dead. No laboratory testing. Necropsy: lesions consistent with trauma. Diagnosis: trauma. | WMO-S | Control (trauma) |
| 49 | Juvenile/ Male | 142 | 0.61 | 7-Apr-99 | Chemehuevi Valley Site 20, San Bernadino County | Clinical: low growth rate, lethargic, variety of scute, scale and shell lesions. Lab: anemia, hypoproteinemia, hypoalbuminemia, low vitamin E and selenium; *Mycoplasma* negative. Necropsy: sunken and irregular scutes; moderate osteopenia of shell and long bones, edema, testicular and retinal degeneration, mild rhinitis. Diagnosis: malnutrition | NCO | Malnutrition |
| 50 | Adult/ Female | 195 | 1.55 | 27-Apr-99 | Chemehuevi Valley Site 20, San Bernadino County | Clinical: low growth rate, lethargic, variety of scute, scale and shell lesions. Lab: anemia, hypoproteinemia, hypoalbuminemia, low vitamin E and selenium; *Mycoplasma* negative. Necropsy: scute and scale lesions, multifocal epidermal epithelial necrosis, subacute dermatitis with heterophils, lymphocytes, and bacterial and fungal colonization, mild osteopenia of shell, shell necrosis with heterophilic inflammation, bacteria, and fungal hyphae; cardiac muscle degeneration; mild rhinitis. Diagnosis: 1) cutaneous fungal and bacterial infection, 2) malnutrition | NCO | Infection/inflammation  Other: malnutrition |
| 51 | Juvenile/ Male | 160 | 0.88 | 13-May-99 | Chemehuevi Valley Site 20, San Bernadino County | Clinical: low growth rate, lethargic, variety of scute, scale and shell lesions. Lab: anemia, hypoproteinemia, hypoalbuminemia, low vitamin E and selenium; *Mycoplasma* negative. Necropsy: lesions of cutaneous dyskeratosis on plastron and scales; mild osteopenia, hepatocellular vacuolation, testicular degeneration, and thyroid atrophy; mild rhinitis. Diagnosis: 1) cutaneous dyskeratosis 2) malnutrition. | NCO | Cutaneous dyskeratosis  Other: malnutrition |
| 54 | Adult/ Female | 183 | 1.26 | 12-Apr-00 | Fenner Valley, San Bernadino County | Clinical: fair body condition with shell lesions. Lab: mild monocytosis, high bile acids, low vitamin A and selenium; *Mycoplasma* and herpesvirus negative. Necropsy: minimal subcutaneous fat, muscle mass reduced by 25-50%, multifocal advanced lesions of cutaneous dyskeratosis with bone necrosis, fungal colonization, dermatitis with bacteria and yeast, and osteopenia; internal organ and skeletal and cardiac muscle atrophy. Diagnosis: 1) severe cutaneous dyskeratosis; 2) bacterial and fungal dermatitis; 3) loss of condition | EMO | Cutaneous dyskeratosis  Other: infection/ inflammation and loss of condition |
| 55 | Adult/ Male | 240 | 2.24 | 28-Apr-00 | Fenner Valley, San Bernadino County | Clinical: severe shell lesions, reduced muscle mass. Laboratory: anemia, mild monocytosis, increased granular lymphocytes, increased ALP, hypoproteinemia, hypoalbuminemia, hypokalemia, high bile acids, low vitamin E and selenium; *Mycoplasma* negative; positive for herpesvirus. Necropsy: shell and skin lesions consistent with cutaneous dyskeratosis, with osteopenia, fungal colonization, and dermatitis with intralesional bacteria; thyroid dysplasia; minimal subcutaneous fat, muscle mass reduced 25%, atrophy of internal organs and skeletal and cardiac muscle, mild rhinitis. Diagnoses: 1) multifocal cutaneous dyskeratosis with bacterial infection, 2) loss of condition, 3) herpesvirus. | EMO | Cutaneous dyskeratosis  Other: infection/ inflammation, loss of condition |
| 56 | Adult/ Male | 235 | 2.2 | 9-May-00 | Fenner Valley, San Bernadino County | Clinical: reduced muscle mass, shell lesions. Lab: lymphocytosis, monocytosis, mild azotemia, high CK and bile acids, low vitamin A, vitamin E and selenium; concentrated urine; *Mycoplasma* and herpesvirus negative. Necropsy: shell lesions consistent with cutaneous dyskeratosis, with osteopenia and dermatitis, a few fungal colonies cultured from scute; fat gelatinous and absent subcutaneously, muscle mass reduced 25%, *Sarcocystis* sp. noted. Diagnoses: 1) Multifocal cutaneous dyskeratosis with osteopenia and dermatitis; 2) loss of condition, 3) *Sarcocystis* sp. | EMO | Cutaneous dyskeratosis  Other: loss of condition |
| 57 | Adult/ Male | 295 | 3.8 | 6-Jun-00 | Sand Hills, Marine Corps Air Ground Combat Center, San Bernadino County | Clinical: wet nose, swollen eyes, low muscle mass, shell wear. Lab: high CK and bile acids; *Mycoplasma* serology and culture positive; negative for herpesvirus. Necropsy: muscle mass reduced by 25-50%, edema, mild dermatitis, mild pneumonia, hepatic and testicular atrophy, splenic hemosiderosis, moderate skeletal and cardiac muscle atrophy. Diagnosis: 1) mycoplasmosis, 2) mild cutaneous dyskeratosis, 3) loss of condition | WMO-S | Mycoplasmosis  Other: loss of condition |

*Details of necropsy findings for 35 of the tortoises can be found in Homer et al., 1998, and Berry et al., 2002.

ECO, Eastern Colorado; NECO, Northern Colorado; EMO, East Mojave; WMO-W, West Mojave-West; WMO-C, West Mojave-Central; WMO-S, West Mojave-South

**SupplementaryTable 2.** Wilcoxon post hoc pairwise comparisons (see Table 4) for element concentrations in Agassiz’s desert tortoises (*Gopherus agassizii*) based on region in the Mojave and Colorado (western Sonoran) deserts.

| **Tissue** | **Element** | **Significant Wilcoxon Pairwise Comparisons** | **P Value** |
| --- | --- | --- | --- |
| Keratin | Hg | EMO < COLO  EMO < WMO-S | 0.010  0.009 |
|  | Na | WMO-WC > COLO  WMO-WC > EMO | 0.019  0.034 |
|  | Se | EMO > COLO  EMO > WMO-S  EMO > WMO-WC | 0.040  0.029  0.003 |
| Kidney | Cd | WMO-WC < COLO  WMO-WC < EMO  WMO-WC < WMO-S | 0.011  0.004  0.011 |
|  | Cu | EMO > WMO-S  EMO > WMO-WC | 0.047  0.009 |
| Liver | Cd | WMO-WC < EMO  WMO-WC < WMO-S | 0.022  0.008 |
|  | Fe | WMO-WC < COLO  WMO-WC < EMO  WMO-WC < WMO-S  EMO > COLO | 0.032  0.005  0.032  0.023 |
|  | K | WMO-WC < COLO | 0.009 |
|  | Mo | WMO-WC < EMO  WMO-WC < WMO-S  WMO-S < COLO | 0.037  0.020  0.035 |
|  | Se | EMO > WMO-WC  WMO-S > WMO-WC | 0.028  0.006 |
|  | V | WMO-WC < EMO  WMO-WC < WMO-S | 0.041  0.017 |

COLO = Colorado desert; EMO = East Mojave desert; WMO-S = West Mojave desert, southern area; WMO-WC = West Mojave desert, west and central areas

**SupplementaryTable 3.** Wilcoxon post hoc pairwise comparisons (see Table 5) for element concentrations in Agassiz’s desert tortoises (*Gopherus agassizii*) based on primary disease process.

| **Tissue** | **Element** | **Significant Wilcoxon Pairwise Comparisons** | **P Value** |
| --- | --- | --- | --- |
| Keratin | Se | Cutaneous dyskeratosis > Trauma  Cutaneous dyskeratosis > Infection/inflammation  Cutaneous dyskeratosis > Malnutrition | 0.003  0.045  0.023 |
|  | Zn | Mycoplasmosis > Infection/inflammation  Trauma > Urolithiasis | 0.044  0.036 |
| Kidney | Ca | Trauma > Cutaneous dyskeratosis  Trauma > Infection/inflammation  Urolithiasis > Infection/inflammation  Infection/inflammation > Cutaneous dyskeratosis | 0.047  0.047  0.032  0.042 |
|  | Cd | Cutaneous dyskeratosis > Trauma  Mycoplasmosis > Trauma  Mycoplasmosis > Malnutrition  Cutaneous dyskeratosis > Malnutrition | 0.032  0.049  0.012  0.015 |
|  | Fe | Trauma > Cutaneous dyskeratosis  Trauma > Infection/inflammation  Trauma > Mycoplasmosis | 0.014  0.009  0.008 |
|  | Zn | Malnutrition > Trauma | 0.010 |
| Liver | Ca | Trauma > Cutaneous dyskeratosis  Trauma > Infection/inflammation  Urolithiasis > Cutaneous dyskeratosis | 0.018  0.020  0.045 |
|  | Cd | Cutaneous dyskeratosis > Trauma  Cutaneous dyskeratosis > Infection/inflammation  Urolithiasis > Trauma  Urolithiasis > Cutaneous Dyskeratosis  Mycoplasmosis > Trauma | 0.014  0.048  0.047  0.045  0.018 |
|  | Cu | Cutaneous dyskeratosis >Trauma  Cutaneous dyskeratosis > Infection/inflammation  Malnutrition > Trauma  Malnutrition > Infection/inflammation  Urolithiasis > Trauma | 0.013  0.011  0.015  0.018  0.032 |
|  | Fe | Cutaneous dyskeratosis > Trauma  Infection/inflammation > Trauma  Malnutrition > Trauma  Mycoplasmosis > Trauma  Urolithiasis > Cutaneous dyskeratosis | 0.002  0.022  0.007  0.014  0.045 |
|  | Na | Malnutrition > Trauma  Malnutrition > Infection/inflammation | 0.046  0.023 |
|  | Se | Cutaneous dyskeratosis > Trauma  Urolithiasis > Trauma  Mycoplasmosis > Trauma | 0.003  0.032  0.018 |
|  | Zn | Cutaneous dyskeratosis > Trauma  Malnutrition > Trauma  Urolithiasis > Trauma | 0.011  0.008  0.047 |

**Supplementary Table 4**. Agassiz’s desert tortoises (*Gopherus agassizii*) with outlying high concentrations of trace minerals, macrominerals and heavy metals, salvaged between 1993 and 2000 from the Mojave and Colorado deserts of California, USA.

| **Element** | **Method of Outlier**  **Identification** | **Keratin** | **Kidney** | **Liver** |
| --- | --- | --- | --- | --- |
| Al | Box plot  (>1.5X IQR) | 272 ppm (#31) | 204 ppm (#35)  38 ppm (#34)  22 ppm (#42)  16 ppm (#36) | 370 ppm (#33)  66 ppm (#57) |
| As | Rare detectables  (4/118, 3.4%) | 1. ppm (#32)   11 ppm (#19) | 1.9 ppm (#6) | 1.9 ppm (#12) |
| Au | Rare detectables  (8/92, 8.7%) | 5.1 ppm (#38)  4.6 ppm (#20)  0.3 ppm (#37) | 2.0 ppm (#37)  1.8 ppm (#38) | 1.8 ppm (#37)  0.6 ppm (#33)  1.0 ppm (#34) |
| B | Box plot  (>1.5X IQR) | 15.4 ppm (#54) | — | — |
| Ba | Box plot  (>1.5X IQR) | 7.9 ppm (#49, Juvenile)  7.1 ppm (#50)  6.1 ppm (#46, Juvenile) | 3.7 ppm (#36)  3.0 ppm (#35)  2.5 ppm (#39)  1.9 ppm (#24)  1.1 ppm (#23) | 7.8 ppm (#36)  5.6 ppm (#33)  4.3 ppm (#24)  1.4 ppm (#10)  1.1 ppm (#39)  1.0 ppm (#27) |
| Ca | Box plot  (>1.5X IQR) | 5900 ppm (#50)  4300 ppm (#41) | 1650 ppm (#36)  1300 ppm (#24)  1200 ppm (#43)  970 ppm (#35)  900 ppm (#39) | 1600 ppm (#36)  870 ppm (#26, Juvenile)  780 ppm (#24)  680 ppm (#33)  530 ppm (#23)  450 ppm (#25, Juvenile) |
| Cd | Box plot  (>1.5X IQR) | 3.5 ppm (#54) | 5.3 ppm (#24)  5.2 ppm (#57)  5.2 ppm (#12) | 2.3 ppm (#23)  1.8 ppm (#19) |
| Co | Box plot  (>1.5X IQR) | — | 1.2 ppm (#49, Juvenile) | — |
| Cr | Box plot  (>1.5X IQR) | 1.3 ppm (#47)  0.8 ppm (#45) | 0.7 ppm (#23) | 7.1 ppm (#37)  3.2 ppm (#28) |
| Cu | Box plot  (>1.5X IQR) | — | 13 ppm (#39)  11 ppm, (#56)  6.6 ppm (#45) | 75 ppm (#22)  63 ppm (#26, Juvenile)  40 ppm (#55)  30 ppm (#57)  30 ppm (#10) |
| Fe | Box plot  (>1.5X IQR) | 350 ppm (#31) | 220 ppm (#35) | 3300 ppm (#18)  3000 ppm (#20) |
| Hg | Box plot  (>1.5X IQR) | 0.50 ppm (#40)  0.26 ppm (#45)  0.19 ppm (#46, Juvenile) | 1.15 ppm (#56)  0.76 ppm (#55) | — |
| K | Box plot  (>1.5X IQR) | 4803 ppm (#48)  896 ppm (#22)  739 ppm (#26, Juvenile)  617 ppm (#25, Juvenile) | — | — |
| Mg | Box plot  (>1.5X IQR) | 250 ppm (#25, Juvenile)  230 ppm (#22) | 470 ppm (#43)  300 ppm (#36)  280 ppm (#24) | 370 ppm (#25, Juvenile)  340 ppm (#33)  300 ppm (#26, Juvenile)  280 ppm (#24) |
| Mn | Box plot  (>1.5X IQR) | 7.8 ppm (#24) | 8.3 ppm (#35)  3.6 ppm (#45)  3.2 ppm (#43) | 15.0 ppm (#33)  3.3 ppm (#45)  2.8 ppm (#36) |
| Mo | Box plot  (>1.5X IQR) | — | 3.6 ppm (#19) | 5.1 ppm (#24)  5.0 ppm (#57) |
| Na | Box plot  (>1.5X IQR) | 1700 ppm (#26, Juvenile)  1100 ppm (#25, Juvenile) | 4700 ppm (#23) | 3700 ppm (#25, Juvenile) |
| Ni | Rare detectables  (12/117, 6.6%) | 200 ppm (#54)  4.9 ppm (#47)  2.0 ppm (#45)  2.1 ppm (#50) | 7.5 ppm (#39)  0.9 ppm (#17) | 3.0 ppm (#47)  3.0 ppm (#28)  1.9 ppm (#17)  0.9 ppm (#18)  0.9 ppm (#19)  0.7 pm (#45) |
| P | Box plot  (>1.5X IQR) | 2000 ppm (#41) | — | 4000 ppm (#15) |
| Pb | Rare detectables  (8/122, 6.6%) | 2.6 ppm (#57)  1.5 ppm (#8, Juvenile)  1.5 ppm (#22, Juvenile) | 3.8 ppm (#21, Juvenile)  0.7 ppm (#29) | 2.1 ppm (#21, Juvenile)  1.2 ppm (#16, Juvenile)  0.5 ppm (#37) |
| Se | Box plot  (>1.5X IQR) | 2.4 ppm (#54)  2.4 ppm (#55)  1.8 ppm (#50)  1.6 ppm (#51, Juvenile)  1.1 ppm (#57)  1.0 ppm (#56) | 2.0 ppm (#40) | 3.5 ppm (#50)  2.1 ppm (#51, Juvenile)  2.0 ppm (#55)  1.5 ppm (#54)  1.5 ppm (#57) |
| Sn | Rare detectables  (4/64, 6.2%) | 9.0 ppm (#18)  1.1 ppm (#8, Juvenile)  0.6 ppm (#20) | — | 0.2 ppm (#25, Juvenile) |
| V | Box plot  (>1.5X IQR) | — | 2.4 ppm (#41)  1.4 ppm (#19)  0.9 ppm (#57) | 5.1 ppm (#57)  3.7 ppm (#6)  2.7 ppm (#41)  2.0 ppm (#10) |
| Zn | Box plot  (>1.5X IQR) | 78 ppm (#43) | 230 ppm (#39)  210 ppm (#21, Juvenile)  130 ppm (#16, Juvenile)  71 ppm (#12)  57 ppm (#22, Juvenile)  51 ppm (#45) | 140 ppm (#23)  98 ppm (#21, Juvenile)  86 ppm (#16, Juvenile) |

All results are on a wet weight basis.

**Supplementary Table 5**. A comparison of examples of elemental toxicants in liver, kidney, and keratin present in Agassiz’s desert tortoise (*Gopherus agassizii*) and in other species of

turtles. Data for Al, As, Ba, Co, Cr, Mn, Ni, Tl, and V are from Table 1. Data for Cd, Cu, Fe, and Zn in kidneys and Cd, Cu, Fe, Hg, Se, and Zn of livers of diseased Agassiz’s desert tortoise,

*Gopherus agassizii,* are from Table 6.

| **Element** | **Species** | **Units^7^** | **Keratin** | **Kidney** | **Liver** | **Reference** |
| --- | --- | --- | --- | --- | --- | --- |
| Al | *Gopherus agassizii* | wwt, ppm | Scute: 93 (18–272) | 3 (1–204) | 11 (1–370) | Present study^1^ |
| Al | *Caretta caretta* | wwt,µg/g |  | 1.06 ± 0.25 | 1.13 ± 0.61 | Yipel et al. (2017)^2^ |
| Al | *Chelonia mydas* | wwt, µg/g |  | 0.80 ± 0.50 | 0.96 ± 0.63 | Yipel et al. (2017)^2^ |
| As | *Gopherus agassizii* | wwt, ppm | Scute: 1.0 (0.1–15.0) | 0.5 (0.1–6.0) | 0.5 (0.1–3.0) | Present study^1^ |
| As | *Caretta caretta* | wwt, µg/g |  | 1.99 ± 1.66 | 1.87 ± 1.45 | Yipel et al. (2017)^2^ |
| As | *Chelonia mydas* | wwt, µg/g |  | BDL^5^ | BDL | Yipel et al. (2017)^2^ |
| As | *Caretta caretta* | wwt, µg/g | Scute: 0.96 (0.05–6.03) |  |  | Miguel et al. (2022)^3^ |
| As | *Caretta caretta* | wwt, µg/g | Scute:1.01 (0.10–2.39) |  |  | Miguel et al. (2022)^3^ |
| As | *Caretta caretta* | dwt, µg/g |  | 0.272 ± 0.014 | 0.94 ± 0.01 | Abdallah (2023)^2^ |
| As | *Chelonia mydas* | dwt, mg/kg |  |  | 0.55 ± 0.75 | Tanabe et al. (2022)^2^ |
| As | *Chelonia mydas* | wwt, mg/kg | Claw: 22.7 ±7.6 SE | 3.37 ± 0.61 | 2.71 ± 0.25 | Faust et al. (2014)^4^ |
| Ba | *Gopherus agassizii* | wwt, ppm | Scute: 1.9 (0.1–7.9) | 0.3 (0.1–3.7) | 0.3 (0.1–7.8) | Present study^1^ |
| Ba | *Chelonia mydas* | wwt, mg/kg | Claw: 7.12 ±1.90 SE | 0.201 ± 0.084 | 0.056 ± 0.003 | Faust et al. (2014)^4^ |
| Cd | *Gopherus agassizii* | wwt, ppm | Scute: 0.1 (0.1–3.6) | 1.3 (0.1–5.3) | 0.5 (0.1–2.3) | Present study^1^ |
| Cd | *Caretta caretta* | wwt, µg/g |  | 4.71 ± 1.66 | 2.01 ± 0.42 | Yipel et al. (2017)^2^ |
| Cd | *Chelonia mydas* | wwt, µg/g |  | 4.24 ± 1.01 | 0.54 ± 0.31 | Yipel et al. (2017)^2^ |
| Cd | *Caretta caretta* | wwt, µg/g | Scute: 0.004, (0.001–0.02) |  |  | Miguel et al. (2022)^3^ |
| Cd | *Caretta caretta* | wwt, µg/g | Scute: 0.008 (0.0002–0.05) |  |  | Miguel et al. (2022)^3^ |
| Cd | *Caretta caretta* | dwt, µg/g |  | 42.49 ± 11.61 | 6.45 ± 3.95 | Abdallah (2023)^2^ |
| Cd | *Chelonia mydas* | wwt, µg/g |  | 5.1 (2.2–7.5) | 4.3 (2.2–9.2) | Storelli et al. (2008)^4^ |
| Cd | *Chelonia mydas* | dwt, mg/kg |  |  | 0.01 ± 0.02 | Tanabe et al. (2022)^2^ |
| Cd | *Chelonia mydas* | wwt, mg/kg | BDL^5^ | 3.07 ± 0.61SE | 0.904 ± 0.127 | Faust et al. (2014)^4^ |
| Co | *Gopherus agassizii* | wwt, ppm | Scutes: 0.1 (0.1–2.5) | 0.2 (0.1–1.2) | 0.1 (0.1–0.3) | Present study^1^ |
| Co | *Chelonia mydas* | dwt, mg/kg |  |  | 0.11 ± 0.08 | Tanabe et al. (2022)^2^ |
| Co | *Chelonia mydas* | wwt, mg/kg | BDL | 0.595 ± 0.057 | 0.273 ± 0.065 | Faust et al. (2014)^4^ |
| Cr | *Gopherus agassizii* | wwt, ppm | Scute: 0.24 (0.03–2.5) | 0.15 (0.05–0.66) | 0.20 (0.05–7.10) | Present study^1^ |
| Cr | *Caretta caretta* | wwt, µg/g |  | 0.15 ± 0.06 | 0.16 ± 0.09 | Yipel et al. (2017)^2^ |
| Cr | *Chelonia mydas* | wwt, µg/g |  | 0.05 ± 0.05 | 0.04 ± 0.05 | Yipel et al. (2017)^2^ |
| Cr | *Caretta caretta* | wwt, µg/g | Scute: 0.5 (0.01–3.27) |  |  | Miguel et al. (2022)^3^ |
| Cr | *Caretta caretta* | wwt, µg/g | Scute: 0.39 (0.01–1.81) |  |  | Miguel et al. (2022)^3^ |
| Cr | *Chelonia mydas* | dwt, mg/kg |  |  | 10.29 ± 8.35 | Tanabe et al. (2022)^2^ |
| Cr | *Chelonia mydas* | wwt, mg/kg | Claw: 84.3 ± 29.4 | 4.28 ± 0.18 | 4.35 ± 0.11 | Faust et al. (2014)^4^ |
| Cu | *Gopherus agassizii* | wwt, ppm | Scute: 0.1 (0.1–2.1) | 2.3 (0.1–13.0) | 8.4 (0.9–75.0) | Present study^1^ |
| Cu | *Caretta caretta* | wwt, µg/g |  | 1.89 ± 1.26 | 3.14 ± 2.58 | Yipel et al. (2017)^2^ |
| Cu | *Chelonia mydas* | wwt, µg/g |  | 2.03 ± 1.64 | 2.13 ± 1.95 | Yipel et al. (2017)^2^ |
| Cu | *Caretta caretta* | wwt, µg/g | Scute: 0.73 (0.11–2.49) |  |  | Miguel et al. (2022)^3^ |
| Cu | *Caretta caretta* | wwt, µg/g | Scute: 0.99 (0.21–3.20) |  |  | Miguel et al. (2022)^3^ |
| Cu | *Chelonia mydas* | wwt, µg/g |  | 8.2 (4.8–14.3) | 32.8 (18.5–59.0) | Storelli et al. (2008)^4^ |
| Cu | *Chelonia mydas* | dwt, mg/kg |  |  | 10.41 ± 9.77 | Tanabe et al. (2022)^2^ |
| Cu | *Chelonia mydas* | wwt, ppm | Claw: 0.941 ± 0.118 | 3.24 ± 1.11 | 37.1 ± 7.3 | Faust et al. (2014)^4^ |
| Fe | *Gopherus agassizii* | wwt, ppm | Scute: 97 (32–350) | 23 (14–110) | 850 (1990–3300) | Present study^1^ |
| Fe | *Caretta caretta* | wwt, µg/g |  | 36.8 ± 4.79 | 478.75 ± 46.24 | Yipel et al. (2017)^2^ |
| Fe | *Chelonia mydas* | wwt, µg/g |  | 22.10 ± 2.59 | 297.6 ± 43.16 | Yipel et al. (2017)^2^ |
| Fe | *Caretta caretta* | wwt, µg/g | Scute: 358 (29.9–2621) |  |  | Miguel et al. (2022)^3^ |
| Fe | *Caretta caretta* | wwt, µg/g | Scute: 247 (48.9–863) |  |  | Miguel et al. (2022)^3^ |
| Fe | *Chelonia mydas* | dwt, mg/kg |  |  | 423.58 ± 111.89 | Tanabe et al. (2022)^2^ |
| Hg | *Gopherus agassizii* | wwt, ppm | Scute: 0.03 (0.01–0.50) | 0.15 (0.01–1.15) | 0.28 (0.04–0.82) | Present study^1^ |
| Hg | *Caretta caretta* | wwt, µg/g |  | 0.06 ± 0.05 | 0.07 ± 0.05 | Yipel et al. (2017)^2^ |
| Hg | *Chelonia mydas* | wwt, µg/g |  | 0.03 ± 0.02 | 0.04 ± 0.02 | Yipel et al. (2017)^2^ |
| Hg | *Caretta caretta* | wwt, µg/g | Scute: 0.01 (0.0006–0.54) |  |  | Miguel et al. (2022)^3^ |
| Hg | *Caretta caretta* | wwt, µg/g | Scute: 0.005 (0.0007–0.03) |  |  | Miguel et al. (2022)^3^ |
| Hg | *Caretta caretta* | dwt, µg/g |  | 0.88 ± 0.34 | 1.16 ± 0.76 | Abdallah (2023)^2^ |
| Hg | *Mauremys l. leprosa; M. l. saharica.* | dwt, µg/g | Claw: 1.76 ± 2.308 (0.03–13.956) |  |  | Slimani et al (2018) |
| Mn | *Gopherus agassizii* | wwt, ppm | Scute: 2.1 (0.6–7.8) | 1.1 (0.4–8.3) | 1.0 (0.2–15.0) | Present study^1^ |
| Mn | *Caretta caretta* | wwt, µg/g |  | 0.72 ± 0.15 | 3.38 ± 0.86 | Yipel et al. (2017)^2^ |
| Mn | *Chelonia mydas* | wwt, µg/g |  | 0.31 ± 0.06 | 1.42 ± 0.40 | Yipel et al. (2017)^2^ |
| Mn | *Caretta caretta* | wwt, µg/g | Scute: 8.44 (2.62–24.8) |  |  | Miguel et al. (2022)^3^ |
| Mn | *Caretta caretta* | wwt, µg/g | Scute: 7.16 (2.08–18.4) |  |  | Miguel et al. (2022)^3^ |
| Mn | *Chelonia mydas* | wwt, mg/kg |  |  | 3.11 ± 0.82 | Tanabe et al. (2022)^2^ |
| Mn | *Chelonia mydas* | wwt, mg/kg | Claw: 4.22 ± 1.76 | 1.27 ± 0.17 | 2.31 ± 0.18 | Faust et al. (2014)^4^ |
| Ni | *Gopherus agassizii* | wwt, ppm | Scute: 0.5 (0.5–200) | 0.25 (0.15–7.5) | 0.25 (0.15–7.5) | Present study^1^ |
| Ni | *Caretta caretta* | wwt, µg/g |  | 3.59 ± 3.09 | 5.23 ± 3.31 | Yipel et al. (2017)^2^ |
| Ni | *Chelonia mydas* | wwt, µg/g |  | 3.31 ± 1.69 | 3.98 ± 3.13 | Yipel et al. (2017)^2^ |
| Ni | *Chelonia mydas* | dwt, mg/kg |  |  | 5.6 ± 5.05 | Tanabe et al. (2022)^2^ |
| Ni | *Chelonia mydas* | wwt, mg/kg | Claw: 0.003 ± 0.001 | 0.066 ± 0.007 | 0.151 ± 0.032 | Faust et al. (2014)^4^ |
| Pb | *Gopherus agassizii* | wwt, ppm | Scute: 0.50 (0.05–7.50 | 0.25 (0.15–3.8) | 0.25 (0.15–2.1) | Present study^1^ |
| Pb | *Caretta caretta* | wwt, µg/g |  | 0.07 ± 0.05 | 0.11 ± 0.06 | Yipel et al. (2017)^2^ |
| Pb | *Chelonia mydas* | wwt, µg/g |  | 0.03 ± 0.01 | 0.03 ± 0.02 | Yipel et al. (2017)^2^ |
| Pb | *Caretta caretta* | wwt, µg/g | Scute: 0.05 (0.000006–0.11) |  |  | Miguel et al. (2022)^3^ |
| Pb | *Caretta caretta* | wwt, µg/g | Scute: 0.05 (0.0004–0.34) |  |  | Miguel et al. (2022)^3^ |
| Pb | *Chelonia mydas* | dwt, ppm |  | 6.51 ± 2.12 | 6.12 ± 1.22 | Abdullah (2023)^2^ |
| Se | *Gopherus agassizii* | wwt, ppm | Scute: 0.2 (0.1–2.4) | 0.7 (0.1–2.0) | 0.6 (0.1–3.5) | Present study^1^ |
| Se | *Caretta caretta* | wwt, µg/g |  | 5.98 ± 4.06 | 7.52 ± 4.31 | Yipel et al. (2017)^2^ |
| Se | *Chelonia mydas* | wwt, µg/g |  | BDL | 0.46 ± 0.34 | Yipel et al. (2017)^2^ |
| Se | *Trachemys s. scripta* | dwt, ppm |  | 4.38 ± 0.12 | 2.04 ± 0.12 | Haskins et al. (2017)^4,6^ |
| Se | *Chelonia mydas* | dwt, mg/kg |  |  | 2.53 ± 0.72 | Tanabe et al. (2022)^2^ |
| Se | *Chelonia mydas* | wwt, ppm | Claw: 2.96 ± 0.82 | 0.881 ± 0.089 | 1.65 ± 0.15 | Faust et al. (2014)^4^ |
| Tl | *Gopherus agassizii* | wwt, ppm | Scute: <15 | <5.0 | <5.0 | Present study^1^ |
| Tl | *Chelonia mydas* | wwt, mg/kg | BDL | BDL | BDL | Faust et al. (2014)^4^ |
| V | *Gopherus agassizii* | wwt, ppm | Scute: 0.3 (0.1–2.5) | 0.2 (0.1–2.4) | 0.3 (0.1–5.1) | Present study^1^ |
| V | *Chelonia mydas* | wwt, mg/kg | Claw: 23.6 ± 8.8 | 1.17 ± 0.02 | 1.24 ± 0.02 | Faust et al. (2014)^4^ |
| Zn | *Gopherus agassizii* | wwt, ppm | Scute: 40 (16–84) | 28 (16–230) | 34 (9–140) | Present study^1^ |
| Zn | *Caretta caretta* | wwt, µg/g |  | 9.47 ± 2.23 | 14.46 ± 6.56 | Yipel et al. (2017)^2^ |
| Zn | *Chelonia mydas* | wwt, µg/g |  | 3.82 ± 2.44 | 4.04 ±1.85 | Yipel et al. (2017)^2^ |
| Zn | *Caretta caretta* | wwt, µg/g | Scute: 33.7 (5.90–86.9) |  |  | Miguel et al. (2022)^3^ |
| Zn | *Caretta caretta* | wwt, µg/g | Scute: 34.1 (3.59–97.9) |  |  | Miguel et al. (2020)^3^ |
| Zn | *Chelonia mydas* | wwt, µg/g |  | 26.4 (14.6–38.5) | 34.5 (19.3–53.9) | Storelli et al. (2008)^4^ |
| Zn | *Chelonia mydas* | dwt, mg/kg |  |  | 36.57 ± 6.51 | Tanabe et al. (2022)^2^ |
| Zn | *Chelonia mydas* | wwt, mg/kg | Claw: 146 ± 15 | 27.9 ± 5.7 | 35.0 ± 3.3 | Faust et al. (2014)^4^ |

^1^Data expressed as median (minimum-maximum)

^2^Data expressed as mean ± SD

^3^Data expressed as mean (minimum-maximum) or range

^4^Data expressed as mean ± SE

^5^BDL: Below detection limits of the method used

^6^Only the controls are shown

^7^ppm = µg/g = mg/kg
